# Repeated Evolution Versus Common Ancestry: Sex Chromosome Evolution in the Haplochromine Cichlid *Pseudocrenilabrus philander*

**DOI:** 10.1093/gbe/evz003

**Published:** 2019-01-12

**Authors:** Astrid Böhne, Alexandra Anh-Thu Weber, Jelena Rajkov, Michael Rechsteiner, Andrin Riss, Bernd Egger, Walter Salzburger

**Affiliations:** 1 Department of Environmental Sciences, Zoological Institute, University of Basel, Switzerland; 2 Museums Victoria, Melbourne, Victoria, Australia; 3 Program Man Society Environment, University of Basel, Switzerland

**Keywords:** sex determination, species complex, teleost fish, speciation, genome sequencing, population genetics

## Abstract

Why sex chromosomes turn over and remain undifferentiated in some taxa, whereas they degenerate in others, is still an area of ongoing research. The recurrent occurrence of homologous and homomorphic sex chromosomes in distantly related taxa suggests their independent evolution or continued recombination since their first emergence. Fishes display a great diversity of sex-determining systems. Here, we focus on sex chromosome evolution in haplochromines, the most species-rich lineage of cichlid fishes. We investigate sex-specific signatures in the *Pseudocrenilabrus philander* species complex, which belongs to a haplochromine genus found in many river systems and ichthyogeographic regions in northern, eastern, central, and southern Africa. Using whole-genome sequencing and population genetic, phylogenetic, and read-coverage analyses, we show that one population of *P. philander* has an XX–XY sex-determining system on LG7 with a large region of suppressed recombination. However, in a second bottlenecked population, we did not find any sign of a sex chromosome. Interestingly, LG7 also carries an XX–XY system in the phylogenetically more derived Lake Malawi haplochromine cichlids. Although the genomic regions determining sex are the same in Lake Malawi cichlids and *P. philander*, we did not find evidence for shared ancestry, suggesting that LG7 evolved as sex chromosome at least twice in haplochromine cichlids. Hence, our work provides further evidence for the labile nature of sex determination in fishes and supports the hypothesis that the same genomic regions can repeatedly and rapidly be recruited as sex chromosomes in more distantly related lineages.

## Introduction

Sexual reproduction is nearly universal across eukaryotes (Speijer et al. 2015; Garg and Martin 2016). One of the most puzzling aspects of this ancient trait is the remarkable contrast between ultra-conserved features (e.g., meiosis, ploidy changes, and cell fusion) and plastic components (e.g., sex determination and modes of reproduction) (Lode 2012; Heule et al. 2014; Capel 2017; Pannell 2017). In particular, the great diversity of sex-determining (SD) mechanisms suggests their repeated and continuous evolution throughout the eukaryotic tree of life (reviewed by Heitman [2015], Blackmon et al. [2017], Capel [2017], and Pannell [2017]), supporting the view of sex as a threshold phenotype that can be canalized into either one of two discrete states by a variety of extrinsic or intrinsic factors as well as a combination thereof (Perrin 2016; Capel 2017). The involvement of extrinsic factors in SD is summarized under the term environmental sex determination (ESD). Intrinsic factors, commonly referred to as genetic sex determination (GSD), comprise systems ranging from single base pair differences between the sexes (Kamiya et al. 2012) to highly differentiated sex chromosomes as in mammals or birds (Graves 2006, 2008, 2014) and including polyfactorial SD (Moore and Roberts 2013) and even SD via RNA instead of protein-coding genes (Akagi et al. 2014; Kiuchi et al. 2014). Sex chromosomes originate from autosomes when one locus acquires a mutation such that heterozygous individuals develop into one sex, whereas homozygous ones develop into the other sex. If sex chromosomes evolve within an ancestrally hermaphroditic (or monoecious) species, at least two mutations are necessary to induce the evolution of GSD (Muller 1932; Westergaard 1958; Charlesworth and Charlesworth 1978) and hence of sex chromosomes.

The canonical model of sex chromosome evolution predicts that suppression of recombination between such proto-sex chromosomes is favored (Muller 1918) and adjacent sexually antagonistic mutations may cause the spread of reduced recombination along the chromosome (Charlesworth [2017] but see also Cavoto et al. [2018]). Suppressed recombination will lead to a reduced effective population size of the sex-limited chromosome (Y in male-heterogametic species; W in female-heterogametic species) and an increase of Hill–Robertson interferences (Charlesworth et al. 1987; Charlesworth and Charlesworth 2000; Charlesworth 2017). Deleterious mutations on the Y/W can no longer be purged and, consequently, accumulate under the impact of Muller’s ratchet, background selection, and selective sweeps (Charlesworth and Charlesworth 2000; Charlesworth et al. 2005). This can lead to chromosomal decay, as exemplified by the mammalian Y chromosome (reviewed by Graves [2006], Bellott and Page [2009], and Schartl et al. [2016]). One escape route to this “evolutionary trap” can be sex chromosome turnover suggested to be induced by deleterious mutation load (Blaser et al. 2013) or sex-antagonistic mutations occurring on autosomes (van Doorn and Kirkpatrick 2007; van Doorn and Kirkpatrick 2010) driving the evolution of a new sex chromosome pair. Sex chromosome turnovers have indeed been shown in, for example, fishes and amphibians (Miura 2007; Volff et al. 2007; Kitano and Peichel 2012; Sessions et al. 2016; Jeffries et al. 2018), with cichlids illustrating the role of sexual antagonism as a driving force in this process (Roberts et al. 2009).

Alternatively, low levels of recombination might be maintained between the two sex chromosomes that are sufficient to allow the purging of deleterious mutations from Y or W chromosomes (Guerrero et al. 2012; Dufresnes et al. 2014). The loci that pave the way for sex chromosome evolution are often unknown. Still, comparisons across different animal taxa revealed the recurrent evolution of certain genes as master SD genes. This has led to the proposition that there are “limited options” for SD genes or even sex chromosomes (Marshall Graves and Peichel 2010).

With ∼3,000–4,000 species, cichlid fishes are one of the largest vertebrate families (Salzburger and Meyer 2004). Because of their taxonomic richness, their phenotypic and ecologic diversity, and their propensity to diversify, cichlids are an important model system in evolutionary biology (Kornfield and Smith 2000; Henning and Meyer 2014; Seehausen 2015; Salzburger 2018). The most species-rich lineage within Cichlidae is Haplochromini, which includes the members of the adaptive radiations in Lakes Victoria and Malawi (together ∼1,200 species), many riverine and lacustrine species elsewhere in Africa (Turner et al. 2001; Verheyen et al. 2003; Schwarzer et al. 2009; Schwarzer et al. 2012), as well as ∼30 species endemic to Lake Tanganyika (the “Tropheini”) (Salzburger et al. 2005).

**Fig. 1.**
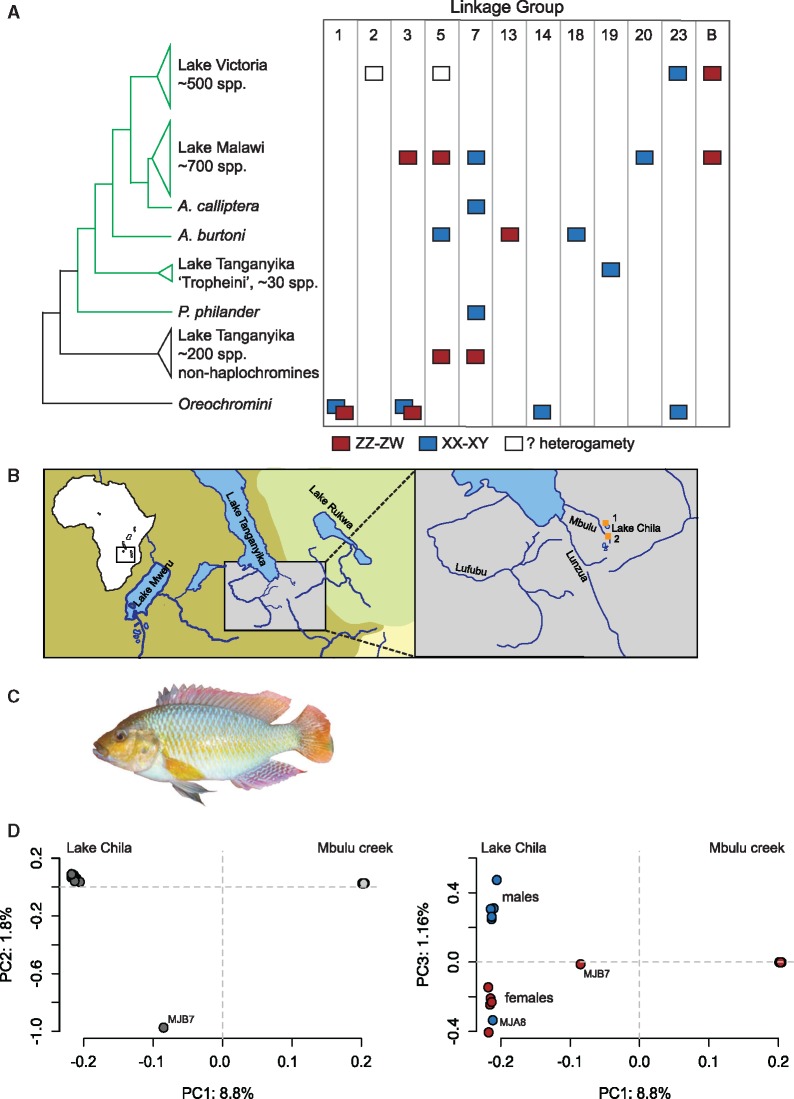
—Phylogenetic relationships and sex determination in East African cichlids. (*A*) Schematic phylogenetic relationships of East African cichlids. Information on sex determination systems based on Böhne et al. (2016), Cnaani et al. (2008), Feulner et al. (2018), Gammerdinger et al. (2018a, b), Kudo et al. (2015), Parnell and Streelman (2013), Peterson et al. (2017), Roberts et al. (2016), Ser et al. (2010), and Yoshida et al. (2011). Haplochromine lineages are depicted in green. (*B*) Map of East Africa and a zoom on the sampling locations: 1, Lake Chila and 2, Mbulu creek. (*C*) Male specimen of *Pseudocrenilabrus philander.* (*D*) PCA on genome-wide variant data of all *P. philander* individuals of this study. PC1 separates the lake individuals from the creek population. PC3 separates males from females. The outlier MJB7 and the potential sex-reversed individual MJA8 are highlighted: dark gray: Lake Chila, light gray: Mbulu creek, red: females, and blue: males.

Cichlid fishes perfectly exemplify the plastic components of sexual reproduction in that closely related species feature various breeding systems and a variety of SD mechanisms including ESD and GSD systems (Römer and Beisenherz 1996; Cnaani et al. 2008; Ser et al. 2010; Yoshida et al. 2011; Parnell et al. 2012; Parnell and Streelman 2013; Reddon and Hurd 2013; Kudo et al. 2015; Böhne et al. 2016; Roberts et al. 2016; Peterson et al. 2017; Feulner et al. 2018; Gammerdinger et al. 2018a, b) (fig. 1*A*). Cichlids are, thus, an excellent model to study the dynamics of SD system evolution. Previous research on the evolution of SD systems in African cichlids lends some support to the “limited options” hypothesis. Two particular chromosomes (corresponding to LG5 and LG7 in the Nile tilapia genome, an outgroup species to the East African Great Lakes, often used as reference) have repeatedly been recruited as sex chromosomes in different species of the East African Great Lakes (Parnell et al. 2012; Kudo et al. 2015; Böhne et al. 2016; Roberts et al. 2016; Peterson et al. 2017; Gammerdinger et al. 2018a; Ser et al. 2010).

In this study, we approach cichlid sex determination from a phylogenetic perspective by investigating sex chromosome signatures in the *Pseudocrenilabrus philander* species complex, a member of a sister-clade to the modern haplochromines of Lakes Victoria, Malawi, and Tanganyika. We sampled two populations for whole-genome sequencing in northern Zambia: Mbulu creek and Lake Chila, a small lake 20 km south of Lake Tanganyika, which is connected to the Mbulu creek via its outflow (fig. 1*B* and *C*). The *P. philander* species complex (Katongo et al. 2005; Koblmuller et al. 2012) comprises two major mitochondrial lineages, one representing the Zambezi–Kafue drainage and one lineage of mainly Congolese origin (Egger et al. 2015). Both lineages occur in Lake Chila, with the Zambezi–Kafue lineage being far more frequent (Egger et al. 2015). Population assignment tests based on microsatellite data suggest that the two lineages represent a single panmictic population. The Mbulu creek population belongs to the Zambezi–Kafue lineage and experienced genetic bottlenecks probably induced by strong seasonal variation in water volume (Egger et al. 2015). Upon the inspection of 24 newly sequenced *P. philander* genomes and a marker-based approach in a larger set of individuals, we provide strong evidence for an XX–XY SD system on LG7 in the lake population. We could not detect this or any other GSD system in the genomes of the creek population. We compare our results to an XX–XY system in the same genomic region of cichlids from Lake Malawi (Ser et al. 2010; Parnell and Streelman 2013; Peterson et al. 2017). Finally, we show that the XX–XY SD system on LG7 in *P. philander* possibly evolved within Lake Chila, because it seems absent in other populations of the *P. philander* species complex.

## Materials and Methods

### Sampling, DNA Extraction, and Sequencing

For this study, we sampled six males and six females of *P. philander* from Lake Chila and 12 individuals (4 males, 3 females, and 5 juveniles) from the adjacent Mbulu creek for whole-genome sequencing (fig. 1). In addition, we included 78 specimens sampled for a previous study (Egger et al. 2015) for polymerase chain reaction (PCR) genotyping (see below). Fin clips and whole specimens were preserved in ethanol. Individuals were sexed by visual inspection of the gonads and body coloration. Five specimens from Mbulu creek did not show distinguishable gonads and were defined as juveniles. DNA was extracted from fin clips with EZNA Tissue DNA Kit (Omega Bio-Tek). Individual genomic libraries were prepared with TruSeq DNA PCR-free Low Sample Kit (Illumina), pooled per population and subsequently sequenced (150 bp paired-end) on four lanes of an Illumina HiSeq3000 by the genomics facility of the D-BSSE (Basel, Switzerland; supplementary table S1, Supplementary Material online). Sequencing data were deposited in the SRA (SRP148476). Research involving animals was performed with approval of the Swiss authorities under a research permit issued by the Lake Tanganyika Research Unit, Department of Fisheries, Mpulungu, Zambia.

### Raw Data Processing, Read Alignment, Variant Calling, and Filtering

Raw reads were inspected with FastQC (0.11.3, https://www.bioinformatics.babraham.ac.uk/projects/fastqc/; last accessed January 23, 2019) and adapters trimmed with Trimmomatic 0.36 (ILLUMINACLIP:TruSeq3-PE-3.fa:2:30:10:2:true) (Bolger et al. 2014). We used the Nile tilapia (*Oreochromis niloticus*) genome assembly version 2 (refseq accession number GCF_001858045.1_ASM185804v2) as reference. Unplaced scaffolds were concatenated lexicographically into an “UNPLACED” super chromosome. This reference was indexed with BWA 0.7.13 and alignments of each individual performed using bwa-mem with default parameters (Li and Durbin 2009) (table 1 and supplementary table S1, Supplementary Material online). Alignments were coordinate sorted and indexed with SAMtools 1.3.1 (Li et al. 2009). We performed an indel realignment (RealignerTargetCreator and IndelRealigner, GATK 3.4.0) (McKenna et al. 2010). Variants were called with GATK’s HaplotypeCaller (per individual and per chromosome), GenotypeGVCFs (per chromosome), and CatVariants (to merge all obtained VCF files). The VCF file was filtered with DP < 100; DP > 800; MQ < 20; FS > 60; SOR > 10; MQRankSum < −10; ReadPosRankSum < −10; and QD < 2. Variants with >50% missing data were excluded using –max-missing 0.5 in VCFtools 0.1.14 (Danecek et al. 2011).

**Table 1:** Detailed Information and Genome Statistics for the Individuals Used in This Study

Sample Name	Population	Mitochondrial Haplotype	Phenotypic Sex	Genotypic Sex	Mean Seq. Coverage	Genome Wide *F*_IS_	All but LG7 *F*_IS_	LG7 *F*_IS_
MJA4	LC	Ht31	F	F	15.32	0.183	0.162	0.450
MJA6	LC	Ht31	F	F	15.15	0.163	0.149	0.341
MJC1	LC	Ht31	F	F	13.56	0.226	0.219	0.312
MJC2	LC	Ht31	F	F	13.49	0.229	0.219	0.360
MJC3	LC	Ht18	F	F	14.14	0.223	0.211	0.381
MJB7	LC	Ht32	F	F	14.34	−0.345	−0.338	−0.433
MJA8	LC	Ht18	M	F	13.14	0.239	0.226	0.420
MJB1	LC	Ht31	M	M	12.95	0.219	0.239	−0.038
MJB3	LC	Ht31	M	M	12.86	0.201	0.219	−0.038
MJB5	LC	Ht18	M	M	13.38	0.185	0.209	−0.121
MJB8	LC	Ht31	M	M	13.44	0.242	0.263	−0.019
MJB9	LC	Ht31	M	M	14.10	0.169	0.189	−0.081
MJC7	MC	Ht13	M	U	13.66	0.166	0.165	0.187
MJC8	MC	Ht13	M	U	15.00	0.097	0.095	0.139
MJC9	MC	Ht13	M	U	12.71	0.208	0.205	0.266
MJE7	MC	Ht13	M	U	14.73	0.095	0.087	0.237
MJD1	MC	Ht13	F	U	16.39	0.048	0.045	0.101
MJD2	MC	Ht13	F	U	13.47	0.181	0.172	0.320
MJD3	MC	Ht13	J	U	13.15	0.219	0.212	0.337
MJD5	MC	Ht13	J	U	13.59	0.182	0.182	0.188
MJD6	MC	Ht13	J	U	14.09	0.178	0.167	0.359
MJD8	MC	Ht13	J	U	14.60	0.153	0.147	0.241
MJD9	MC	Ht13	J	U	15.26	0.108	0.100	0.236
MJE6	MC	Ht13	F	U	13.45	0.179	0.175	0.250

Note.—Mitochondrial haplotypes correspond to naming in Egger et al. (2015). Mean sequencing coverage was calculated on the final VCF file. *F*_IS_ was calculated on the final VCF file subset per population. LC, Lake Chila; MC, Mbulu creek; F, female; M, male; J, juvenile; U, undifferentiated; *F*_IS_, inbreeding coefficient.

### Population Structure and Phylogeny

To assess population structure, between-population genome-wide *F*_ST_, average *d*_*xy*_ (absolute divergence), and average *π* (nucleotide diversity) were calculated in 10 kb windows on the filtered VCF file including single nucleotide polymorphisms (SNPs) and indels using evo (https://github.com/millanek/evo/; last accessed January 23, 2019). Average *d*_a_ (net divergence) was calculated using Nei and Li’s formula: *d*_a_ = *d_xy_* – (*π_x_* + *π_y_*)/2 (Nei and Li 1979). Tajima’s *D* was calculated for each population in 10 kb windows in VCFtools 0.1.14 (Danecek et al. 2011). Population structure was examined on the whole-genome VCF data set with a principal component analysis (PCA) using smartPCA (Eigensoft 6.1.4) (Patterson et al. 2006). Alignments to the mitochondrial reference scaffold NC_013663.1 were extracted from individual BAM files, sorted with SAMtools 1.3.1. (Li et al. 2009) and converted to fastq format using Picard 2.8.0 SamToFastq (http://broadinstitute.github.io/picard; last accessed January 23, 2019). Mitochondrial genomes were reconstructed from these reads with MIRA 4 (Chevreux et al. 1999). The regions corresponding to the control region (D-Loop) were subsequently extracted and aligned with additional public sequences from the *P. philander* species complex (sequences from Egger et al. [2015]); using MAFFT online service 7 (Katoh et al. 2017) under the FFT-NS-i option, that is, with fast construction of an initial alignment followed by iterative refinement until convergence. Identical sequences were collapsed into haplotypes using DNA collapser (FaBox) (Villesen 2007). Bayesian inference of phylogeny was done in MrBayes 3.2.2 (Ronquist et al. 2012). Posterior probabilities were obtained from Markov chain Monte Carlo simulations in two independent runs (10 chains with 10 Mio generations each, chain temperature: 0.25, trees sampled every 1,000 generations) using the best-fit model of molecular evolution as suggested by jModelTest (Posada 2008). A 50% majority-rule consensus tree was constructed after a 1 million generation burn-in (chain stationarity and run parameter convergence were checked with Tracer 1.6, http://tree.bio.ed.ac.uk/software/tracer/; last accessed January 23, 2019, using posterior probability as a measure of clade support). A whole nuclear genome phylogeny was built by reconstructing for each individual a sequence corresponding to the first haplotype of each linkage group using samtools faidx (LG) (Li et al. 2009) and bcftools consensus –haplotype 1 (BCFtools 1.5, https://samtools.github.io/bcftools/; last accessed January 23, 2019). The sequences of each linkage group were then concatenated and merged into one sequence per individual using EMBOSS union (Rice et al. 2000). Maximum likelihood inference was done with RAxML 8.2.11 (–*k*, −# 100, −*f**a*) (Stamatakis 2014). Branch length estimation (−*k*) is given in number of mutations per bp per generation. In order to obtain divergence times in number of generations, we used the Lake Malawi cichlid mutation rate estimation of 3.5 × 10^−9^ per bp per generation (95% CI: 1.6 × 10^−9^ to 4.6 × 10^−9^) from Malinsky et al. 2018. The VCF file was phased and genotypes were imputed with Beagle 4.1 (Browning and Browning 2007, 2016). For topology weighting, we used *Twisst* (Martin and Van Belleghem 2017) with 1, 5, and 10 kb windows to infer if Chila and Mbulu males were more closely related to each other than to the females of their respective population in a specific region of LG7.

### Sex Chromosome Identification and Characterization of the Type of SD System on LG7

Male–female *F*_ST_ and difference in nucleotide diversity (*π*_diff_ = *π*_males_ − *π*_females_) were calculated in 10 kb windows on the filtered VCF file including SNPs and indels with evo (https://github.com/millanek/evo/; last accessed January 23, 2019). We tested for a difference in nucleotide diversity between males and females of each population with a Welch two sample *t*-test in R 3.4.2. (R Core Team 2017). We calculated male–female *F*_ST_ per population (five males vs. five females for Lake Chila; four males vs. three females for Mbulu creek) as well as for both populations combined. A maximum likelihood phylogeny was reconstructed as described above on LG7 only and on all chromosomes excluding LG7. A relatedness statistic (unadjusted Ajk statistic) (Yang et al. 2010), of all individuals was calculated separately for LG7 and for all the remaining chromosomes in VCFtools (–relatedness and –chr LG7 or –not-chr LG7). *F*_IS_ (inbreeding coefficient) was calculated separately for LG7 and all LGs excluding LG7 in VCFtools (–het and –chr LG7 or –not-chr LG7) for each individual within its respective population. The inbreeding coefficient *F*_IS_ was also calculated in 10 kb windows per sex within each population along LG7 and correlated to male–female *F*_ST_ following the method described by Rodrigues and Dufresnes (2017). To obtain the average normalized *F*_IS_ value per sex for each 10 kb window, the per individual genome-wide *F*_IS_ value excluding LG7 was subtracted from the LG7 individual *F*_IS_ value. Then, the individual normalized *F*_IS_ values were averaged per sex. Next, we selected biallelic sites from the initial filtered, unphased VCF file for five males and five females from the lake population of the same mitochondrial linage resulting in a total of 30,811,926 sites. We selected sites for which all females were homozygous and all five males heterozygous (XY-sites) as proposed by Brelsford et al. (2017). XY-sites on LG7 were annotated using SnpEff 4.3 (Cingolani et al. 2012).

### De Novo Genome Assemblies and Alignment

We followed the pipeline described in Malmstrøm et al. (2017) to generate a female and male draft genome de novo assembly for Lake Chila *P. philander* using CeleraAssembler 8.3 (Myers et al. 2000) and FLASh 1.2.11 (Magoc and Salzberg 2011), pooling the raw reads of three females and three males. Assembly quality was assessed with QUAST 4.5 (Gurevich et al. 2013) and assembly completeness with BUSCO 3 (Simao et al. 2015) (supplementary table S2, Supplementary Material online). To anchor contigs onto the *O. niloticus* reference genome, we used LAST 861 (lastdb –uNEAR –cR11; lastal –m75 –E0.05) (Kiełbasa et al. 2011). MAF alignment output was converted into tabular format with LAST. Female alignments to LG7 were extracted from the tabular output and filtered to keep scaffolds of >2 kb length and alignment sequence coverage of 50% resulting in 3,340 contigs representing the X chromosome. Scaffolds were ordered based on the start position of their longest alignment. For comparative purposes, we extracted the female scaffolds aligning to LG6 with the same settings (2,048 contigs).

### Sequence Coverage Analysis

Coverage was calculated for each sex from mapping against the de novo assembled genomes. Quality filtered reads of the five male and five female individuals of Lake Chila were mapped against the female and male draft genome using bwa-mem of BWA (Li and Durbin 2009). Alignments were converted to BAM format, sorted, and indexed with SAMtools 1.3.1 (Li et al. 2009). Coverage per individual per site was calculated with samtools depth –aa (SAMtools 1.3.1) (Li et al. 2009). The median coverage against the female de novo assembly over all sites and all individuals per sex for each population was calculated in R 3.3.1, resulting in 17 for Lake Chila males and 18 for Lake Chila females. We did the same analysis keeping only alignments with zero mismatches resulting in a median coverage of 3 in Lake Chila males and 4 in females. Next, we calculated median coverage per site and sex for the scaffolds anchored to LG7 and LG6 (for comparative reasons) and normalized it by the sex-specific median. From these values, we calculated averages of 10 kb windows, which were log 2 transformed for plotting. These steps were run in R using the packages reshape 0.8.7 (Wickham 2007), miscTools 0.6-22 (Henningsen and Toomet 2016), zoo 1.8 (Zeileis and Grothendieck 2005), and ggplot2 2.2.1 (Wickham 2009). From the mapping against the male de novo assembly, we identified regions of “male-only-coverage” (potential Y-specific sequences) as regions in which consecutive positions of 1 kb length had coverage in at least four out of the five males with a total coverage >5 and a coverage over all females <3.

### K-mer Analysis and Assemblies

To assemble Y chromosome–specific sequences, we followed a method described by Akagi et al. (2014). We identified Y-specific reads over their difference in k-mer composition compared with female reads. Raw reads were filtered with Trimmomatic 0.36 (Bolger et al. 2014) (PE mode, adapters.fasta.2:30:10LEADING:3TRAILING:3SLIDINGWINDOW:4:15MINLEN:5). From the trimmed reads, we generated k-mer tables for all 37 k-mers starting with the trigger sequence “AG” and having at least 5 counts reducing the k-mer complexity and computational cost as established by Akagi et al. (2014) using a Python script provided by the Comai lab. Using “CT” as the trigger sequence yielded similar results (data not shown). For comparative reasons, we applied the same method to a human data set of Great Britain ancestry from the 1000 Genomes Project Consortium (http://www.internationalgenome.org/; last accessed January 23, 2019, samples ERR020230, ERR050089, SRR189815, ERR050086, SRR068180, and SRR190845) that had already been used in a k-mer assembly for Y chromosomes (Carvalho and Clark 2013).

Resulting male and female k-mer counts were compared and potential Y-k-mers identified as k-mers that had >9 counts in males but <5 counts in females resulting in 3,612,202 unique Y-k-mers (out of 130,094,951 total unique k-mers). We extracted male reads matching these Y-k-mers and their mate with bbduk (BBTools 37.57, https://jgi.doe.gov/data-and-tools/bbtools/; last accessed January 23, 2019).

The resulting 55,627,673 read pairs were de novo assembled with MEGAHIT 1.1.1 (Li et al. 2015) with stepsize 10, kmin 21, kmax 121, and minimum length 1 kb. Male and female reads were back-mapped on the so-obtained 122,977 contigs. We removed contigs that had over 50 reads coverage at a single position in at least one male (likely individual specific repetitive elements) and those with a 5 read coverage in females. The resulting 233 contigs were blasted against the male and female draft genomes (Blast+ 2.6.0, BlastN with -qcov_hsp_perc 70 and -num_alignments 10, all other settings in default) (Camacho et al. 2009), and discarded if they had a match to the female genome with ≥95% sequence identity. From these remaining 138 contigs, 35 were also present in the full male genome assembly. The 138 contigs were loaded into Blast2GO (Conesa et al. 2005) and scanned for coding sequences with the integrated version of AUGUSTUS (Hoff and Stanke 2013) and *Danio rerio* as reference organism. Obtained genes were blasted against nr (BlastX), searched against Interpro, mapped, and annotated with default settings within Blast2GO. We calculated male and female coverage for these contigs following the same method as described for the stringent method of X-chromosomal coverage.

### K-mer Composition of the X Chromosome

To extract k-mers from the X chromosome, we selected k-mers that had a female/male count ratio between 1.75 and 2.25. The obtained 7,227,218 k-mers were blasted against the reconstructed X chromosome of *P. philande*r with BlastN-short allowing only for perfect matches and maximum 10 alignments per query (Blast+ 2.6.0) (Camacho et al. 2009) resulting in 424,156 k-mers placed on the X chromosome.

### Comparison of LG7 in Other Cichlids

LG7 carries an XY system in cichlids from Lake Malawi. WGS sequences for *Astatotilapia calliptera*, *Aulonocara stuartgranti*, and *Lethrinops lethrinus* were downloaded from the SRA (accession numbers in supplementary table S3, Supplementary Material online), transformed to fastq, trimmed, quality filtered, and mapped to the Nile tilapia genome as described above. Variant calling, filtering, and phasing were also performed as described above. For each individual (24 *P. philander* and 6 from Lake Malawi), a sequence corresponding to the first haplotype of LG7 was extracted using bcftools consensus –haplotype 1 BCFtools 1.5 (https://github.com/samtools/bcftools; last accessed January 23, 2019). Maximum likelihood inference and subsequent divergence time estimation were performed as described above. To infer if *P. philander* males and Lake Malawi males were more closely related in a specific region of LG7 than to their respective females, fixed-length phylogenies were calculated with *Twisst* (Martin and Van Belleghem 2017) using 1, 5, and 10 kb window sizes. For each window size, the support for each topology was quantified by counting the number of windows supporting strongly (100% data; >75% data) or moderately (>66% data) each topology. For comparative purposes, the same topology weighting analysis was also performed on LG6.

We extracted reads aligning to the genomic region of *gsdf* plus 2 kb up- and down-stream (*O. niloticus*: NC_031972.1: 17,568,814–17,579,211). Alleles per individual of the *gsdf* region were de novo assembled using SeCaPr (Andermann et al. 2018) and maximum likelihood phylogenies were conducted as described above. In the same way, we constructed phylogenies for eight candidate genes of sex determination (supplementary table S7, Supplementary Material online, candidate genes are marked in yellow and genomic coordinates from the reference genome are indicated).

Sex-specific variant sites for Lake Malawi cichlids were retrieved from O’Quin (2014) and visually inspected. Sequences for two XX–XY loci described by Parnell et al. (2012) and Parnell and Streelman (2013) were downloaded from SNPdb and placed on the Nile tilapia genome using Blast (Camacho et al. 2009). Marker 27028 (SNP: rs267732628) is located on scaffold NW_017615339.1: 59,608–59,966. Marker 45045 (SNP: rs267732730) is located on NC_031972.1: 1,010,601–1,010,981. We extracted raw reads corresponding to these regions with SAMtools (Li et al. 2009). BAM files were sorted and indexed using SAMtools (Li et al. 2009). Genotypes of the two SNPs for each individual (24 *P. philander* and 6 Lake Malawi cichlids) were visually inspected using SAMtools tview (Li et al. 2009).

### PCR Genotyping of Lake Chila Y-Chromosomal Markers

DNA was extracted from fin clips preserved in ethanol applying a proteinase K digestion followed by a high-salt extraction (Bruford et al. 1998), or already extracted DNA from Egger et al. (2015) was used. Two potential Y-chromosomal markers (*herc3* and *K02A2.6-like*) were coamplified with the autosomal control gene *rpl7*. Primers for *herc3* (GCAAGAAAAGGCTTGTGAACC, TGACAGATACTGGGAGTGAGA), *K02A2.6-like* (GAAACTGACCTCACAGCCCA, GCCAGAAGTTTGTTTGGCGA), and *rpl7* (TGCGGGATAAAAGCGTTAGGA, ATTCCTTGCAGCAGTCATAGA) were constructed on the Lake Chila male de novo genome assembly using Primer-Blast (https://www.ncbi.nlm.nih.gov/tools/primer-blast/; last accessed January 23, 2019). PCR was performed on 5 ng of DNA in a final volume of 12.5 μl using REDTaq DNA polymerase (Sigma-Aldrich) following the manufacturer’s instructions (annealing temperature 58 °C, 35 PCR cycles) Each PCR was done twice. Amplification was verified on 1.5% Tris-acetate-ethylenediaminetetraacetic-acid agarose gels with SYBR Green (ThermoFisher).

## Results

### Genome-Wide Statistics, Population Structure, and Demography

The 24 individuals sequenced in this study could all be assigned to previously identified mitochondrial DNA (mtDNA) haplotypes and fell into clades described by Egger et al. (2015). All specimens from Mbulu creek and eight Lake Chila specimens featured mtDNA haplotype Ht13 (supplementary fig. S1*A*, Supplementary Material online) (Egger et al. 2015), three Lake Chila specimens had Ht18 of the Kafue–Zambezi lineage; and one Lake Chila sample, MJB7, displayed Ht32 (table 1 and supplementary fig. S1*A*, Supplementary Material online).

Aligning the *P. philander* genome sequences to the *O.niloticus* reference genome resulted in 38,260,972 variant sites (SNPs and indels, table 1). The mean sequencing coverage per individual ranged from 12.7× to 16.4× (table 1) being in a range that allows accurate genotyping of heterozygous sites with the GATK multisample caller (Cheng et al. 2014; Meynert et al. 2014). A whole-genome nuclear phylogeny showed that Lake Chila and Mbulu creek populations are reciprocally monophyletic, with an estimated coalescence time of about 620,000 generations for Mbulu creek (95% CI: 472,000–1,357,000) and 912,000 generations for Lake Chila (95% CI: 694,000–1,996,000, supplementary table S4 and supplementary fig. S1*B*, Supplementary Material online). Genome-wide *F*_ST_ between the two populations was 0.538, average *d*_*xy*_ (absolute genetic divergence) was 0.00764, and average *d*_a_ (net genetic divergence) was 0.00411 (table 2). Lake and creek individuals were clearly separated on PC1 in a genome-wide PCA (fig. 1*D*). The Mbulu creek population displayed low levels of within population nucleotide diversity *π* (0.00193; ∼2.6-fold smaller than Lake Chila) and a highly positive Tajima’s *D* (0.42; ∼8-fold larger than Lake Chila, table 2), indicative of an excess of haplotypes compared with the number of segregating sites, compatible with an ongoing population contraction event, that is, a bottleneck. This reduction in effective population size is further supported by the short branch lengths of the Mbulu individuals in the whole-genome phylogeny (supplementary fig. S1*B*, Supplementary Material online). Finally, the bottleneck scenario for the Mbulu creek population is corroborated by the genome-wide PCA, where all Mbulu individuals are strongly overlapping on the first and second PC axes, as well as by their identical mtDNA haplotypes (table 1, fig. 1*D*, and supplementary fig. S1*A*, Supplementary Material online).

**Table 2:** Genome-Wide Population Statistics for *Pseudocrenilabrus philander* from Lake Chila and Mbulu Creek

Statistic	Population Analysis		
	Lake Chila Versus Mbulu Creek	Within Lake Chila	Within Mbulu Creek
*F*_ST_	0.538	—	—
Mean *d_xy_*	0.00764	—	—
Mean *d*_a_	0.00411		
Mean *π*	—	0.00512	0.00193
Mean Tajima’s *D*	—	0.0515	0.4273

Note.—*F*_ST_, relative divergence; *d_xy_*, absolute divergence; *d*_a_, net divergence; *π*, nucleotide diversity.

One female of the Lake Chila population (MJB7) displayed a negative genome-wide *F*_IS_ (table 1 and supplementary table S5, Supplementary Material online), indicating that its heterozygosity is higher than expected under Hardy–Weinberg equilibrium; furthermore, it belongs to a different mtDNA lineage and is clearly separated from all other individuals in PC2 of the genome-wide PCA (fig. 1*D*). Taken together, this is suggestive of MJB7 being a hybrid between a Lake Chila *Pseudocrenilabrus* individual and an unknown second parent. To avoid any bias potentially induced by the high levels of heterozygosity, MJB7 was excluded from further analyses.

Interestingly, PC3 of the genome-wide PCA separated males and females from Lake Chila (fig. 1*D*). This signal cannot be explained by intralake genetic structure, as males and females share mtDNA haplotypes (table 1 and supplementary fig. S1, Supplementary Material online) and form a dense cluster on the first two PCA axes (fig. 1*D*). One phenotypic male from Lake Chila (MJA8) clustered with the Lake Chila females, suggesting that it is a sex-reversed individual (fig. 1*D*). Therefore, MJA8 was also excluded from further analyses.

### LG7 Functions as a Sex Chromosome in the Lake Population of *P. philander*

**Fig. 2.**
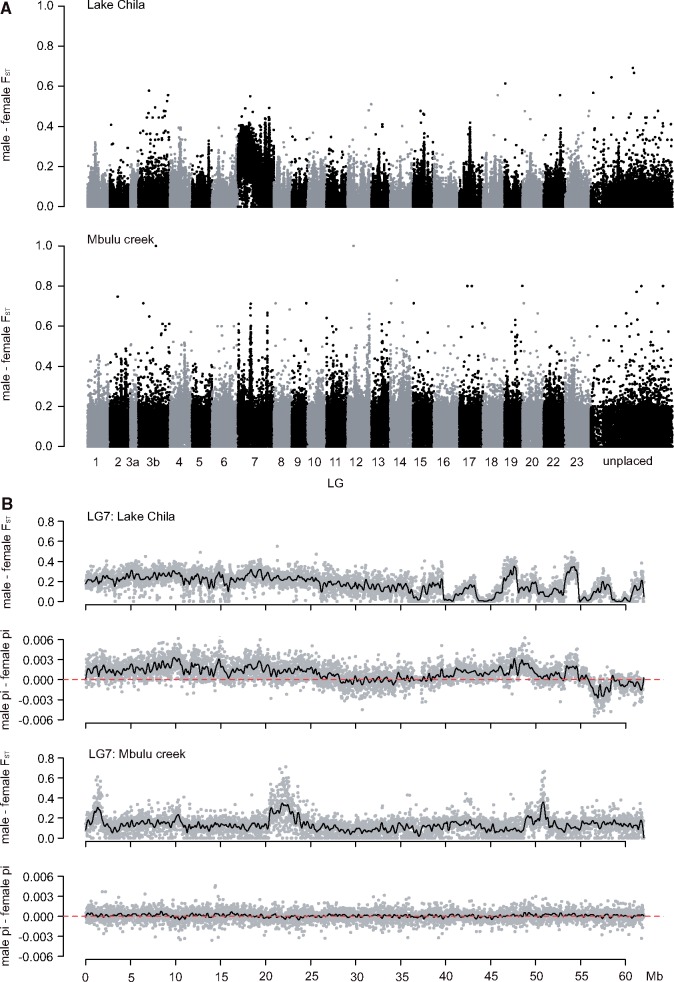
—Genomic signatures of male–female differentiation in *Pseudocrenilabrus philander*. (*A*) Male–female *F*_ST_ for individuals from Lake Chila (upper panel) and Mbulu creek (lower panel) along the reference genome of *Oreochromis niloticus*. Each dot represents a single *F*_ST_ value per 10 kb window. (*B*) Male–female *F*_ST_ and difference in nucleotide diversity between sexes (*π*_diff_ = *π*_males_ − *π*_females_) along LG7. Each gray dot represents a single value per 10 kb window. Black line: smoothed value (loess parameter = 0.01) and red line: no difference in nucleotide diversity between males and females.

**Fig. 3.**
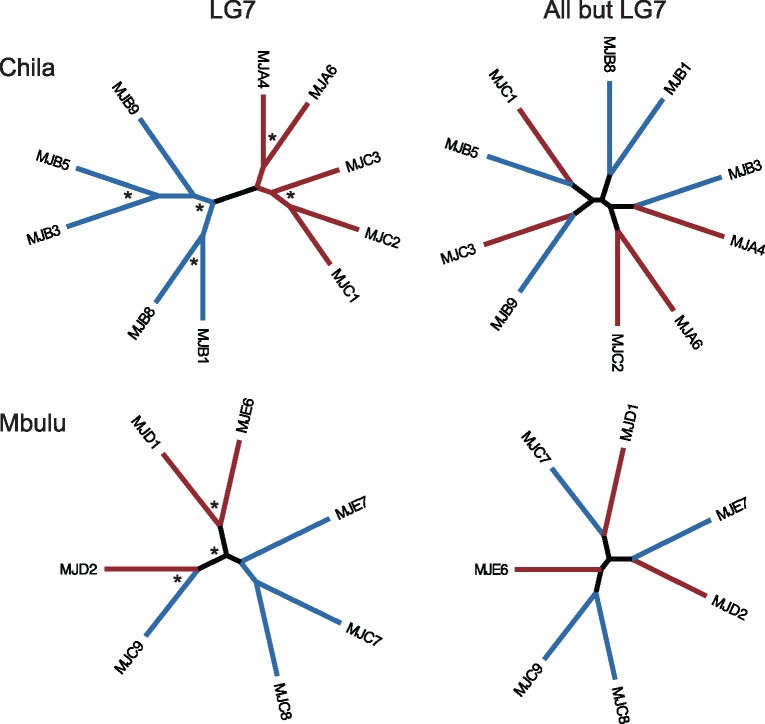
—Phylogenetic analysis within the two *Pseudocrenilabrus philander* populations based on markers on LG7 and using genome-wide variants on all LGs but LG7. Maximum likelihood phylogeny of LG7 and all other LGs except LG7 for Lake Chila (upper panel) and Mbulu creek (lower panel); blue: males, red: females, and asterisks: 100% bootstrap support.

Given the clear-cut separation of males and females in PC3 of the genome-wide PCA (fig. 1*D*), we next aimed to identify the genomic region responsible for the differentiation between the sexes. We first calculated genome-wide *F*_ST_ between males and females within each population and *F*_ST_ per chromosome. The average genome-wide male–female *F*_ST_ within the lake population was 0.04 (average male–female *F*_ST_ excluding LG7: 0.032), whereas the average *F*_ST_ for LG7 was 0.18 indicating a large region of male–female differentiation on this chromosome (fig. 2*A* and supplementary fig. S2, Supplementary Material online). Next, males and females formed distinct clades in a phylogeny on variant data of LG7 only, whereas no such grouping was found when all LGs excluding LG7 were considered (fig. 3), nor in phylogenies built from any other individual LG (supplementary fig. S3, Supplementary Material online). Furthermore, relatedness analyses (Yang et al. 2010) showed that males and females formed two distinct groups on LG7 but did not do so when all linkage groups except LG7 were considered (supplementary fig. S4, Supplementary Material online). Finally, a PCA based on LG7 only, clearly separated males and females from Lake Chila on PC2 (supplementary fig. S5*A*, Supplementary Material online), whereas the first three principal components did not separate the sexes in a PCA based on sequence information from all LGs but LG7 (supplementary fig. S5*B* and *C*, Supplementary Material online).

Contrastingly, in the bottlenecked Mbulu creek fish, the male–female *F*_ST_ of LG7 alone was similar to the genome-wide level (LG7: *F*_ST (male__–__female)_ = 0.12, genome-wide: *F*_ST (male__–__female)_ = 0.09, and genome-wide excluding LG7: *F*_ST (male__–__female)_ = 0.077). However, these values should be taken with caution due to the low sample size of the Mbulu creek population. Furthermore, individuals did not cluster by sex in any of the phylogenies reconstructed from individual LGs (supplementary fig. S3, Supplementary Material online), nor in a LG7 relatedness analysis (supplementary fig. S4, Supplementary Material online). Finally, we performed a topology weighting analysis, using four different “populations”: Lake Chila males, Lake Chila females, Mbulu creek males, and Mbulu creek females. This analysis did not reveal any region where Chila and Mbulu males were more closely related to each other than they were to females (supplementary fig. S6, Supplementary Material online). Therefore, we did not find any evidence for a common sex locus between the two populations.

### LG7 Harbors an XY System in the Lake Population

In a simple sex-chromosomal system, the heterogametic sex shares half of its sex-chromosomal alleles with the homogametic sex (e.g., X alleles in an XX–XY system and Z alleles in a ZZ–ZW system), whereas Y/W alleles are specific to the heterogametic sex. This results in an expected maximum male–female *F*_ST_ of 0.5 for completely sex-differentiated sites (i.e., if the allele frequency for the heterogametic sex is 0.5 and the allele frequency for the homogametic sex is 1, then the expected *F*_ST_ is 0.5 in an infinite population) (Brelsford et al. 2017; Fontaine et al. 2017; Rodrigues and Dufresnes 2017). Furthermore, the heterogametic sex (XY or ZW) shows an excess of heterozygous sites compared with the homogametic sex, reflected by negative *F*_IS_ values. Consequently, *F*_ST_ and *F*_IS_ show a negative correlation in the heterogametic sex (Rodrigues and Dufresnes 2017).

In Lake Chila *P. philander*, males had negative *F*_IS_ values on LG7 (table 1 and supplementary table S5, Supplementary Material online), indicating higher levels of heterozygosity in males. Furthermore, females had higher *F*_IS_ values on LG7 (0.31–0.45) compared with the rest of the genome excluding LG7 (0.15–0.22), denoting low levels of heterozygosity on LG7 (table 1). Males of the lake population also showed significantly higher nucleotide diversity (*π*) compared with females (fig. 2*B* and supplementary fig. S7, Supplementary Material online) and a negative correlation between *F*_IS_ and male–female *F*_ST_ on LG7 (supplementary fig. S8*A*, Supplementary Material online), strongly suggesting that males are the heterogametic sex and that LG7 functions as an XX–XY system.

In the Mbulu creek population, males also displayed higher *π* compared with females (supplementary fig. S7, Supplementary Material online), yet the male–female difference in mean *π* was much smaller than for males and females of Lake Chila (mean *π* Lake Chila males: 0.0039; mean *π* Lake Chila females: 0.0030; mean *π* Mbulu creek males: 0.00103; and mean *π* Mbulu creek females: 0.00094). Moreover, individual *F*_IS_ values did not differ between males and females on LG7. They were higher in both sexes than their corresponding genome-wide estimates, however, overall lower than the female values of the Lake Chila individuals (table 1). Male–female *F*_ST_ on LG7 in the Mbulu population did not indicate a large region with an expected *F*_ST_ for sex chromosomes of 0.5 but several peaks along the chromosome of *F*_ST_ values above 0.5 (fig. 2*A*), which are likely false positives arising from the low sample size. Also, *F*_ST_ and *F*_IS_ along LG7 showed a positive correlation in both sexes (supplementary fig. S8*B*, Supplementary Material online). Hence, there is no indication for an XX–XY system or other sex-specific signals in the Mbulu creek population on any of the LGs.

### Sex Chromosome Differentiation and the SD Region in *P. philander* from Lake Chila

**Fig. 4.**
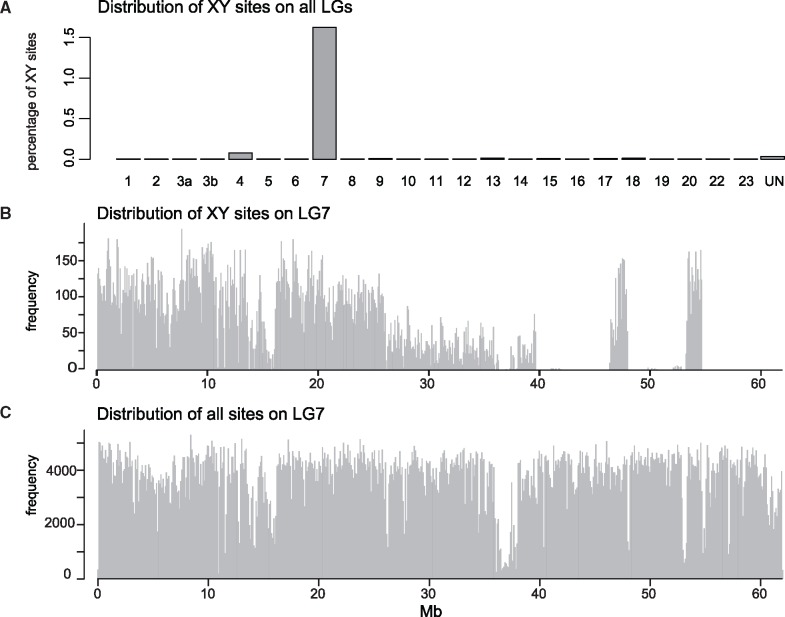
—XY-sites in *Pseudocrenilabrus philander* from Lake Chila. (*A*) Distribution of potential XY sex-patterned sites across all LGs in the Lake Chila population normalized by total number of sites per LG. (*B*) Distribution of XY-sites along LG7 in 10 kb bins. (*C*) Distribution of all variant sites called on LG7 in 10 kb bins.

To further delimit the SD region in Lake Chila fish, we identified sites that showed an XY sex-specific pattern, that is, sites for which all females are homozygous and all males heterozygous. We identified a total of 41,309 XY-patterned sites across the genome, of which the great majority (38,429; 93%) is placed on LG7 (fig. 4*A*). The XY-sites of *P. philander* were distributed along the entire chromosome with a slightly higher frequency at ∼7–12 Mb, (fig. 4*B*) and less to no sites between 27 and 60 Mb with the exceptions of two peaks between 45 and 55 Mb. This block-like distribution of XY-sites might indicate regions of suppressed recombination (e.g., sex chromosome strata) (Lahn and Page 1999), probably caused by chromosomal rearrangements. Alternatively, and probably more likely, these blocks indicate chromosomal rearrangements between *P. philander* and the used reference genome *O. niloticus* and hence a difference in sequence order. The distribution of XY-patterned sites suggests that the SD region is located in the first 25 Mb of LG7. Again, we did not observe such a pattern in the Mbulu creek population (only 2013 potential XY-sites genome-wide, of which 91 are on LG7, supplementary fig. S9, Supplementary Material online).

The number of XY-sites in Lake Chila *P. philander* exceeded that reported for other cichlid sex-chromosomal system. In the Nile tilapia (*O. niloticus*), for example, LG1 has a 9-Mb large XY SD region, which contains 12,225 such sites (out of 38,718 total sex-differentiated sites) (Conte et al. 2017). In the blue tilapia, *Oreochromis aureus*, LG3 carries a ZZ–ZW SD system, which shows 24,983 sex-differentiated sites (total differentiated sites in the genome 103,406) (Conte et al. 2017).

As a next step, we functionally annotated the Lake Chila XY-sites to investigate the effect of variants on coding sequences. The highest density of nonsynonymous sites with “moderate” or “high effect” (i.e., coding sequence variant, frameshift, missense mutation, insertions, deletions, and inversions) was detected between 22 and 23 Mb of LG7 (supplementary fig. S10 and supplementary table S6, Supplementary Material online). The 43 “high effect” variants were located in 33 genes. As expected, Lake Chila males were heterozygous and females homozygous for these SNPs and, all Mbulu creek individuals were homozygous, matching the female lake genotype.

**Fig. 5.**
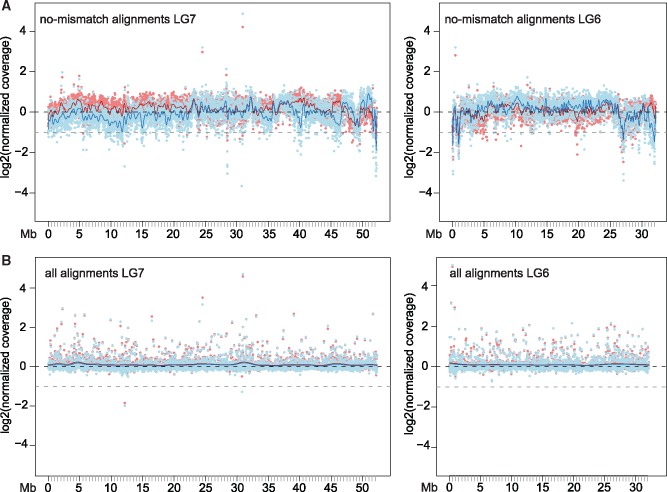
—Sex chromosome coverage in *Pseudocrenilabrus philander* from Lake Chila. (*A*) Coverage of perfect alignments of males (blue) and females (red) along the de novo assembled Lake Chila female X-chromosome (left) and for comparison along the de novo assembled LG6 (right). (*B*) Coverage of all alignments of males and females along the de novo assembled Lake Chila female X-chromosome (left) and for comparison along the de novo assembled LG6 (right); red and blue lines: smoothing spline, black dotted lines: normalized coverage of 1, and gray dotted line: normalized coverage of 0.5.

To further investigate the extent of sex-chromosomal differentiation and delimit the SD region, we analyzed male–female differences in sequence coverage along the sex chromosomes. To avoid any potential bias introduced by using the *O. niloticus* reference genome, we generated a male and a female draft genome assembly for the lake population. In species with heteromorphic XY sex chromosomes, the X chromosome is present in a hemizygous state in males, resulting in ∼50% reduced sequencing coverage for the X in males compared with the X in females or any autosome. When all read alignments with default mapping parameters in the two sexes were considered, which is the standard approach (e.g., Vicoso et al. 2013), no difference in sequence coverage was visible along the X chromosome (fig. 5*B*, coverage follows the expected black line). This indicates that X and Y in *P. philander* from Lake Chila are at early stages of sex chromosome differentiation. However, when considering only perfect alignments (excluding alignments that contain any mismatch), a drop in male sequence coverage became evident, especially in the first 20 Mb of the X chromosome (fig. 5*A*).

**Fig. 6.**
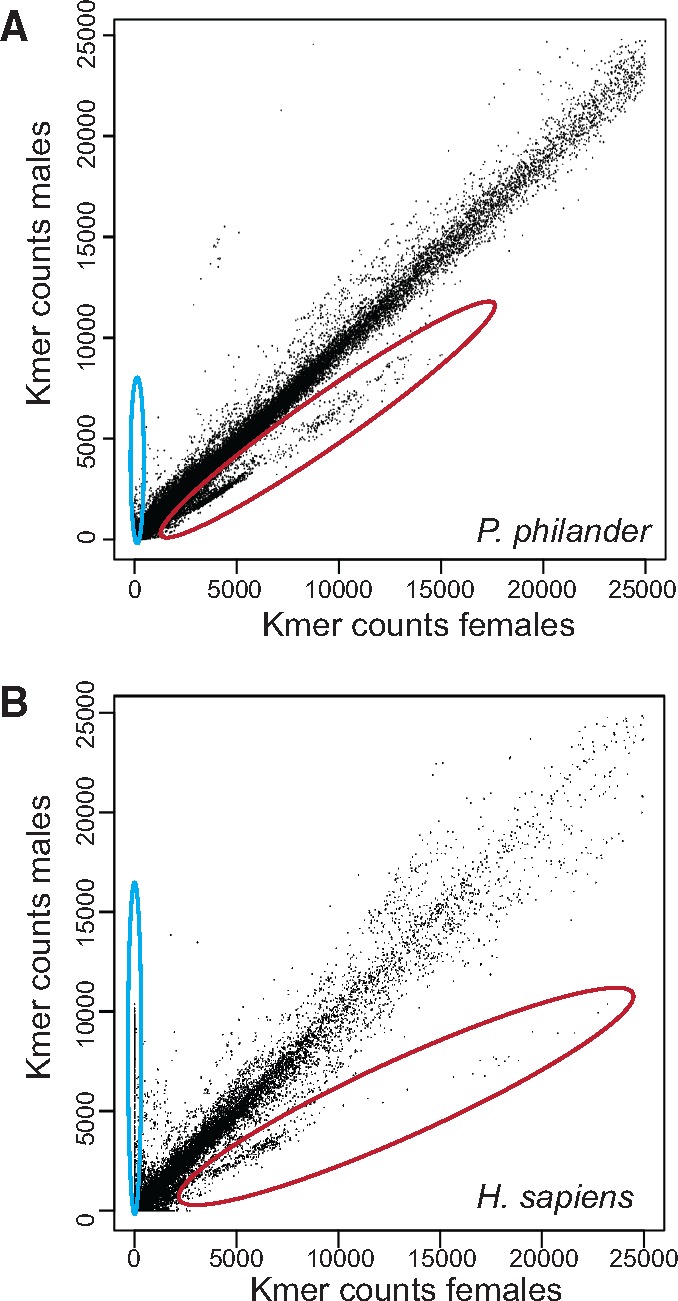
—K-mer comparison in males and females. (*A*) Counts of 37 bp k-mers in male and female Lake Chila *Pseudocrenilabrus philander*. (*B*) Counts of 37 bp k-mers in human males and females. Humans have strongly differentiated sex chromosomes. K-mers derived from the Y chromosome are expected to have zero counts in females; k-mers derived from the X chromosome should have half the count in males than in females. Potential Y-k-mers are highlighted with a blue circle, X-mers with a red circle.

To further investigate this pattern of sex chromosome differentiation, we built a catalog of 37-bp-long subsequences (k-mers) and counted their presence in the male and female reads (fig. 6). Although the sex chromosomes of *P. philander* are certainly much younger and much less differentiated than the one in humans, the k-mer comparison between males and females is similar in these two species (fig. 6). X-linked k-mers are clearly visible in both species as the second largest cloud with higher counts in females than in males (fig. 6, red circle). We investigated the location of potential X-linked k-mers in the female X chromosome assembly which revealed their highest frequency at ∼12.5 Mb (corresponding to ∼15.4 Mb on LG7 in the reference genome, supplementary fig. S11, Supplementary Material online). Combining the analyses of XY-sites, coverage and X-linked k-mers, the SD region *of P. philander* Lake Chila is likely located at 0.3–16 Mb on LG7.

This region has 518 protein-coding gene annotations in the reference genome assembly. A full overview of these genes with corresponding gene ontologies is provided in supplementary table S7, Supplementary Material online, and genes with a potential role in SD are highlighted in yellow. These include two HMG-domain genes, a protein domain also encoded by the mammalian SD gene *Sry* (Sinclair et al. 1990), and *foxl1* and *foxd1*, belonging to the forkhead box family of transcription factors, which play a role in ovarian development and function (Ottolenghi et al. 2005; Uhlenhaut and Treier 2011). They further include *wt1*, which regulates early gonad development in mammals (Wilhelm and Englert 2002).

### Two Reference-Free Approaches to Detect Y-Chromosomal Candidates in the Lake Population

In an XX–XY system, Y chromosome–specific sequences are not present in females resulting in zero sequencing coverage of such regions by female sequencing reads. We searched the male de novo genome assembly for regions of male-only coverage of at least 1 kb in length and detected 12 such regions located on 11 different scaffolds. The longest region was 2,124 bp long. When compared with the reference genome, ten of these scaffolds were placed on LG7 (eight within the first 10 Mb of LG7, supporting the analyses above that this is the SD region) and one on the unplaced scaffold NW_017613955.1. A BlastX search of the candidate regions revealed similarities to five coding sequences (the ubiquitin-protein ligase *herc3* in the 2,124-bp region, two transposable element related sequences, two uncharacterized proteins) and two ncRNAs (supplementary table S8, Supplementary Material online). In the creek population, all but three of these regions showed sequence coverage in both sexes. These three remaining regions, which included the one with *herc3*, do apparently not exist in the creek population genomes (supplementary fig. S12, Supplementary Material online).

Although X and Y are clearly differentiating in Lake Chila *P. philander*, (most of) our analyses revealed a substantial degree of sequence similarity between X and Y and also could not delimit the SD region further than to the first ∼16 Mb of LG7. Our male de novo genome assembly likely contains a consensus assembly for X/Y haplotypes of LG7. When sequencing a male genome of a diploid XY species, Y-specific sequences will have reduced coverage in comparison to autosomal regions. Also, differentiating Y chromosomes typically accumulate repetitive sequences (Chalopin et al. 2015). These two factors may hamper the reconstruction of Y chromosomes using standard assembly tools (Tomaszkiewicz et al. 2017). To identify sequence information derived from Y-specific male-only regions also potentially missing in the reference genome, we applied a method described by Akagi et al. (2014) that makes use of k-mers. We extracted male-specific k-mers from the above-mentioned k-mer catalog and used reads containing them for a targeted assembly of putative Y-chromosomal contigs. We obtained 138 Y-contigs containing 48 potential genes (supplementary table S9, Supplementary Material online), of which 38 could be functionally annotated. Strikingly, 15 of these genes (∼30%) showed strong similarities to transposable elements, suggesting a higher transposable element content on the *P. philander* Y chromosome than the genome-wide average for cichlids of 16–19% (Brawand et al. 2014), a characteristic feature of sex chromosomes (Chalopin et al. 2015).

Among the other genes, we detected two genes involved in spermatogenesis, *psmb2* (Gupta 2005) and *kelch10* (Yan et al. 2004). We also recovered one of the uncharacterized proteins that we previously identified in the full de novo male assembly in a region with zero female coverage (uncharacterized protein *K02A2.6-like*), which functions in nucleic acid and zinc ion binding (supplementary tables S8 and S9, Supplementary Material online). This gene contains a *retropepsinlike* domain of invertebrate retrotransposons (DeMarco et al. 2005).

### LG7 Probably Evolved Twice as a Sex Chromosome in Haplochromine Cichlids

**Fig. 7.**
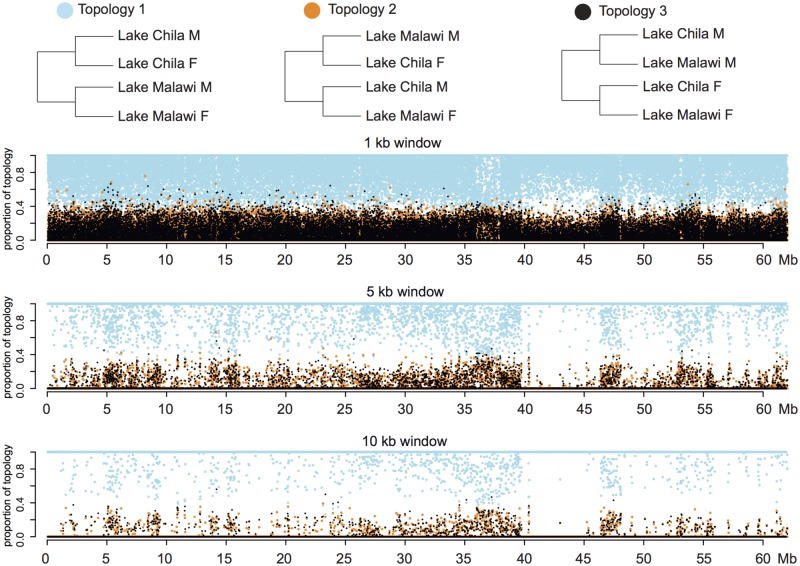
—Topology weighting analysis of LG7. Topology weighting analysis using 1-, 5-, and 10-kb windows between the four “populations” Lake Chila males, Lake Chila females, Lake Malawi males, and Lake Malawi females.

LG7 is known to function as XX–XY system in many haplochromine species endemic to Lake Malawi (Ser et al. 2010; Parnell and Streelman 2013; Peterson et al. 2017) and likely represents the ancestral sex chromosome state of the radiation in this lake (Peterson et al. 2017). We therefore aimed to examine whether or not the Lake Malawi SD system corresponds to the one we identified in *P. philander* of Lake Chila. To this aim, we performed a topology weighting analysis on LG7 to infer if Lake Chila and Lake Malawi males were more closely related to each other compared with the females of their respective population/species in specific genomic regions. If the XX–XY system was ancestral and shared between Lake Malawi cichlids and *P. philander*, one would expect that the SD locus and closely linked loci that do not recombine between X and Y cluster by sex and not by species in a phylogeny (Stock et al. 2011). We included three species (*A.calliptera* XX–XY on LG7 [Peterson et al. 2017]; *A. stuartgranti* and *L.lethrinus*), as these represent, to the best of our knowledge, the only currently available full-genome data covering both sexes per species in Lake Malawi cichlids. Our analyses indicated no strongly supported region in which Lake Malawi and Lake Chila males were more closely related to each other than to the females of their respective species (fig. 7 and supplementary table S10, Supplementary Material online). Rather, the species topology was strongly supported for each window size on LG7, as well as for the non sex-linked LG6 (supplementary fig. S13 and supplementary table S10, Supplementary Material online).

An outstanding candidate gene for the SD locus on LG7 is *gsdf* (*gonadal soma-derived factor*), which has been described as a master SD gene in several fish species (e.g., Myosho et al. 2012; Rondeau et al. 2013). In agreement with this, Peterson et al. (2017) proposed *gsdf* as the SD gene of Lake Malawi cichlids. In another study on Lake Malawi cichlids, focusing on *Metriaclima zebra* and *M. mbenji*, O’Quin (2014) also reported sex-patterned sites in *gsdf*. We thus reconstructed a phylogeny for the *gsdf* locus in the set of Lake Malawi cichlids and *P. philander*. Again, male sequences of the different species did not group together (supplementary fig. S14, Supplementary Material online). When examining the sequences for individual sites, none of them supported a shared sex pattern (supplementary table S11, Supplementary Material online).

In a previous study on the Lake Malawi cichlids *Cynotilapia afra* and *Pseudotropheus elongates*, Parnell and Streelman (2013) detected two distinct XX–XY loci on LG7. We also inspected these XX–XY markers (RAD-tags 27028 and 45045, see figure 4 in Parnell et al. [2012] and Parnell and Streelman [2013]) in *P. philander*. The marker 45045 was homozygous (C/C) in all individuals of all species and all *P. philander*, *A. stuartgranti*, and *L. lethrinus* individuals were homozygous (C/C) for the marker 27028. The *A. calliptera* male was heterozygous (C/T) at this site and the *A. calliptera* female was homozygous (C/C), supporting an XX–XY pattern for this marker only in this species.

Finally, a full LG7 phylogeny including all 24 *P. philander* individuals and male and female individuals from Lake Malawi provides further support for a young age of *P. philander's* sex chromosomes, with a divergence time of ∼423,000 generations for females and ∼455,000 generations for males in LG7 (supplementary fig. S15 and supplementary table S12, Supplementary Material online). Assuming one generation per year, it is reasonable to conclude that X and Y of *P. philander* in Lake Chila diverged less than a million years ago, as the 95% confidence interval did not reach 1 Myr.

We also investigated eight additional single gene phylogenies for genes on LG7 with a potential role in sex determination identified as candidate genes in the SD region of *P. philander* from Lake Chila in this study (supplementary table S7, Supplementary Material online). Similar to *gsdf* and the topology weighting analysis, these gene trees mostly recovered the species tree (supplementary fig. S16, Supplementary Material online). Two genes showed differing topologies, however, with overall low support and not indicative of a shared sex locus.

### LG7 Likely Evolved as a Sex Chromosome within Lake Chila

The LG7 system detected in the Lake Chila population likely evolved independently from the one in Lake Malawi cichlids. Furthermore, we could not detect this system in the adjacent and closely related Mbulu creek population. Given the size of our data set used for full-genome sequencing and the question the origin of this XX–XY system, we aimed to test for the presence/absence of the LG7 XX–XY system in additional individuals of the *P. philander* species complex. We tested 78 individuals belonging to five clades of the *P. philander* species complex and *P. nicholsi* (Egger et al. 2015) by PCR for two markers which were Y chromosome linked in *P. philander* from Lake Chila, namely *herc3* (identified as the largest region absent from the female genomes) and *K02A2.6-like* (also identified as a region with zero female coverage in Lake Chila and over Y-k-mer specific assembly). Within the Lake Chila samples (additional *n* = 34), *herc3* was present in all tested males (15), and 12 males were positive for *K02A2.6-like*. All but two phenotypic females were negative for the two markers. We can thus largely confirm male sex-linkage of the two markers within Lake Chila and hence the presence of an XX–XY SD system in this population. Our PCR assay also included individuals of the two divergent mtDNA haplotype lineages. However, all populations other than Lake Chila did not show sex linkage for the two markers, which were either present or absent in both sexes (supplementary table S13 and supplementary fig. S17, Supplementary Material online).

## Discussion

Cichlid fishes display a breathtaking diversity in basically every phenotypic trait investigated so far including, coloration, morphology, habitat use, breeding systems, or diet (Albertson and Kocher 2006; Sefc 2011; Muschick et al. 2012; Miyagi and Terai 2013; Salzburger 2018) and sex determination is likely another flexible property of this astonishing group of fish. Here, we investigated sex chromosome evolution in a phylogeographically complex species, the haplochromine cichlid *P. philander* (Egger et al. 2015). We detected an XX–XY system in the Lake Chila *P. philander* population, whereas this signature was not detectable in the genomes of an adjacent riverine stock. The creek population likely underwent a genetic bottleneck, so it is possible that the apparent absence of any detectable SD system in this population may be due to demographic events. The creek population may have been founded by XX individuals only, or XY recombination resumed in the creek population. However, markers that were male specific in Lake Chila did not show a sex-specific pattern in specimen from six other *P. philander* populations nor in *P. nicholsi*. Given the nested placement of the Lake Chila population within the *P. philander* species complex (supplementary fig. S1, Supplementary Material online), the most parsimonious explanation for this pattern is that the XX–XY system evolved or at least differentiated within the Lake Chila population.

In agreement with this scenario, we could also not find support for a shared (ancestral) XX–XY LG7 system between Lake Malawi cichlids, and *P. philander* from Lake Chila neither in our divergence time estimates nor in a topology weighting analysis nor in single gene phylogenies for candidate genes of sex determination. We therefore propose that LG7 evolved repeatedly (convergently) as a sex chromosome in different lineages of haplochromine cichlids. This would lend further support to the limited options theory, that is, that certain chromosomes are particularly well suited to become sex chromosomes and evolve as such more often than other chromosomes (Marshall Graves and Peichel 2010). Marshall Graves and Peichel proposed that a likely candidate for a “limited option” is the ancestral teleost chromosome TEL6 (Marshall Graves and Peichel 2010). The sex chromosomes of several fish species are derived from TEL6 including those of the medaka *Oryzias luzonensis*, the sablefish *Anoplomba fimbria* as well as the guppy *Poecilia reticulata* (Marshall Graves and Peichel 2010; Myosho et al. 2012; Rondeau et al. 2013). The marker *SLC45A2* that Marshall Graves and Peichel (2010) used to identify these sex chromosomes as being syntenic to TEL6 is indeed located on LG7 of cichlids (*O. niloticus* LG7: 17,405,957–17,428,885). Together with our study, LG7/TEL6 has been described three times as a sex chromosome in cichlids, suggesting that TEL6 evolved to become a sex chromosome in at least five lineages of teleost fish (Lake Malawi cichlids Ser et al. 2010, Lake Tanganyika cichlid *Hemibates stenosoma*Gammerdinger et al. 2018a, *P. philander* Lake Chila, medaka, guppy, and sablefish) supporting the “limited options” theory. However, other genes on cichlid LG7 are syntenic to TEL 7 (Marshall Graves and Peichel 2010), indicating additional rearrangements of LG7 in cichlids or in the lineage leading towards them. Certainly more data on cichlid sex chromosomes is needed to properly test the “limited options” theory within cichlids.

With our limited data set, we cannot exclude the presence of yet another SD system in the other *P. philander* populations or that the LG7 XX–XY system was present also in the creek but has secondarily been lost or started to recombine again. In addition, in the genome-wide data of the creek population we failed to detect any other sex-chromosomal system. It is possible that a SD region in this population is too small to be detected with our limited sample size. We can also not exclude that this population might rely on an ESD system or a multifactorial combination of environmental and genetic factors. We also report a sex-reversed individual in Lake Chila, as well as mismatches between phenotypic sex and Y chromosome markers in a PCR genotypic assay. If we exclude any sexing errors, this could mean an occurrence of 6–10% of individuals that also do not underlie the XX–XY system within Lake Chila. It could also mean that the markers we tested by PCR genotyping still recombine and are hence not fully Y-linked. Note that the two markers also differed in their presence–absence pattern with *K02A2.6-like* showing only two genotype–phenotype mismatches. It might thus be closer to the actual SD locus than *herc3*. Further (genome-wide) data would be needed to support either of these scenarios.

Still, specimens from the Lake Chila population showed clear signs of sex chromosome differentiation along large sections of LG7, especially in the first 16 Mb. Yet, there were also peaks in male–female *F*_ST_, XY sex–patterned sites as well as male-reduced coverage in other regions along LG7. This block-like distribution of signatures of differentiation (especially visible at 45–50 and 53–55 Mb; figs. 2 and 4) might reflect “sex-chromosome strata,” which are parts of a chromosome that stopped recombining at different points of time in the past (Lahn and Page 1999). This strata formation can result from chromosomal rearrangements such as inversions, which immediately cause suppression of recombination (Sturtevant 1921). Alternatively and probably more likely, these blocks result from genome rearrangements between the reference genome *O. niloticus* and *P. philander*.

We identified several candidate genes for the SD locus in *P. philander* from Lake Chila based on male-specific sequence features. Among these, the most promising ones were *herc3* and the uncharacterized gene *K02A2.6-like*. We would like to point out that *herc3* is also located on the sex chromosomes in another fish, the medaka (Kondo et al. 2006), but is not the master SD gene in this species. We could not find any support for the previously known SD gene *gsdf* as the master SD locus in *P. philander.* The dating of the split between X and Y chromosomes in *P. philander* from Lake Chila to <∼1 Myr suggests a similar age as the one proposed for the origin of the XX–XY system on LG7 in Lake Malawi cichlids (Peterson et al. 2017). When compared with the ZZ–ZW sex determination system on LG3 of another cichlid, the blue tilapia *O. aureus* (Conte et al. 2017), we found that there are more sex-patterned sites in *P. philander* than in *O. aureus*. This suggests a higher level of sex chromosome differentiation in *P. philander*. The LG3 sex chromosome system is ancestral in the Oreochromini lineage (Lee et al. 2004; Cnaani et al. 2008; Cnaani 2013), dating back to the split before *O. aureaus* and *O. niloticus*, estimated to ∼3 Ma (Xiao et al. 2015). Also, our comparison of male–female k-mer compositions in *P. philander* and humans points to a remarkable level of differentiation of the *P. philander* sex chromosomes despite their probably young age.

Autosomes are recruited as sex chromosomes and subsequently follow the path of sex chromosome differentiation as in *P. philander* or the *Oreochromini* lineage. Demographic events such as lake colonizations or population size fluctuations might impact the patterns of differentiation. Under which conditions a differentiated sex chromosome system represents a selective advantage remains an open question, at least for cichlids. Elegant work on sticklebacks demonstrated that newly evolving sex chromosomes contribute to phenotypic divergence and reproductive isolation between sympatric species, probably facilitating speciation (Yoshida et al. 2014). Whether or not the XX–XY system of the Lake Chila individuals causes this population to be reproductively incompatible with other populations remains to be tested. Taken together, our study highlights the contrast between genomic signatures that fit the canonical view on sex chromosome evolution (recombination suppression and sequence differentiation) and the instability that such systems nevertheless face. Remarkably, we show that sex-chromosomal systems can differ within a single cichlid species, at the level of geographically separated populations (see also Böhne et al. [2016]), suggesting that demographic events can impact sex chromosome evolution and, vice versa, that changes in SD systems might contribute to diversification.

## Supplementary Material

Supplementary data are available at *Genome Biology and Evolution* online.

## Supplementary Material

Supplementary Data

## Footnotes

**Data deposition:** The raw sequencing reads were deposited at SRA and are available under the accession SRP148476.

## References

[evz003-B1] Akagi T, Henry IM, Tao R, Comai L. 2014 A Y-chromosome-encoded small RNA acts as a sex determinant in persimmons. Science 346(6209):646–650. 25359977 10.1126/science.1257225

[evz003-B2] Albertson RC, Kocher TD. 2006 Genetic and developmental basis of cichlid trophic diversity. Heredity 97(3):211–221. 16835594 10.1038/sj.hdy.6800864

[evz003-B3] Andermann T, Cano A, Zizka A, Bacon C, Antonelli A. 2018 SECAPR-a bioinformatics pipeline for the rapid and user-friendly processing of targeted enriched Illumina sequences, from raw reads to alignments. PeerJ 6:e5175. 30023140 10.7717/peerj.5175 PMC6047508

[evz003-B4] Bellott DW, Page DC. 2009 Reconstructing the evolution of vertebrate sex chromosomes. Cold Spring Harb Symp Quant Biol. 74:345–353. 20508063 10.1101/sqb.2009.74.048

[evz003-B5] Blackmon H, Ross L, Bachtrog D. 2017 Sex determination, sex chromosomes, and karyotype evolution in insects. J Hered. 108(1):78–93. 27543823 10.1093/jhered/esw047 PMC6281344

[evz003-B6] Blaser O, Grossen C, Neuenschwander S, Perrin N. 2013 Sex-chromosome turnovers induced by deleterious mutation load. Evolution 67(3):635–645. 23461315 10.1111/j.1558-5646.2012.01810.x

[evz003-B7] Böhne A, Wilson CA, Postlethwait JH, Salzburger W. 2016 Variations on a theme: genomics of sex determination in *Astatotilapia burtoni*. BMC Genomics. 17(1):883. 27821061 10.1186/s12864-016-3178-0 PMC5100337

[evz003-B8] Bolger AM, Lohse M, Usadel B. 2014 Trimmomatic: a flexible trimmer for Illumina sequence data. Bioinformatics 30(15):2114–2120. 24695404 10.1093/bioinformatics/btu170 PMC4103590

[evz003-B9] Brawand D, et al 2014 The genomic substrate for adaptive radiation in African cichlid fish. Nature 513(7518):375–381. 25186727 10.1038/nature13726 PMC4353498

[evz003-B10] Brelsford A, Lavanchy G, Sermier R, Rausch A, Perrin N. 2017 Identifying homomorphic sex chromosomes from wild-caught adults with limited genomic resources. Mol Ecol Resour. 17(4):752–759. 27790846 10.1111/1755-0998.12624

[evz003-B11] Browning BL, Browning SR. 2016 Genotype imputation with millions of reference samples. Am J Hum Genet. 98(1):116–126. 26748515 10.1016/j.ajhg.2015.11.020 PMC4716681

[evz003-B12] Browning SR, Browning BL. 2007 Rapid and accurate haplotype phasing and missing-data inference for whole-genome association studies by use of localized haplotype clustering. Am J Hum Genet. 81(5):1084–1097. 17924348 10.1086/521987 PMC2265661

[evz003-B13] Bruford MWO, Hanotte O, Brookfield JFY, Burke T. 1998 Multilocus and single-locus DNA fingerprinting; molecular genetic analysis of populations, a practical approach. Oxford: Oxford University Press.

[evz003-B14] Camacho C, et al 2009 BLAST+: architecture and applications. BMC Bioinformatics 10:421. 20003500 10.1186/1471-2105-10-421 PMC2803857

[evz003-B15] Capel B. 2017 Vertebrate sex determination: evolutionary plasticity of a fundamental switch. Nat Rev Genet. 18(11):675–689. 28804140 10.1038/nrg.2017.60

[evz003-B16] Carvalho AB, Clark AG. 2013 Efficient identification of Y chromosome sequences in the human and *Drosophila* genomes. Genome Res. 23(11):1894–1907. 23921660 10.1101/gr.156034.113 PMC3814889

[evz003-B17] Cavoto E, Neuenschwander S, Goudet J, Perrin N. 2018 Sex-antagonistic genes, XY recombination, and feminized Y chromosomes. J Evol Biol. 31(3):416–427. 29284187 10.1111/jeb.13235

[evz003-B18] Chalopin D, Volff JN, Galiana D, Anderson JL, Schartl M. 2015 Transposable elements and early evolution of sex chromosomes in fish. Chromosome Res. 23(3):545–560. 26429387 10.1007/s10577-015-9490-8

[evz003-B19] Charlesworth B, Charlesworth D. 1978 A model for the evolution of dioecy and gynodioecy. Am Nat. 112(988):975–997.

[evz003-B20] Charlesworth B, Charlesworth D. 2000 The degeneration of Y chromosomes. Philos Trans R Soc Lond B Biol Sci. 355(1403):1563–1572. 11127901 10.1098/rstb.2000.0717 PMC1692900

[evz003-B21] Charlesworth B, Coyne JA, Barton NH. 1987 The relative rates of evolution of sex chromosomes and autosomes. Am Nat. 130(1):113–146.

[evz003-B22] Charlesworth D. 2017 2017. Evolution of recombination rates between sex chromosomes. Philos Trans R Soc Lond B Biol Sci. 372(1736):20160456. 29109220 10.1098/rstb.2016.0456 PMC5698619

[evz003-B23] Charlesworth D, Charlesworth B, Marais G. 2005 Steps in the evolution of heteromorphic sex chromosomes. Heredity 95(2):118–128. 15931241 10.1038/sj.hdy.6800697

[evz003-B24] Cheng AY, Teo YY, Ong RT. 2014 Assessing single nucleotide variant detection and genotype calling on whole-genome sequenced individuals. Bioinformatics 30(12):1707–1713. 24558117 10.1093/bioinformatics/btu067

[evz003-B25] Chevreux B, Wetter T, Suhai S. 1999. Genome sequence assembly using trace signals and additional sequence information. Proceedings of the German Conference on Bioinformatics. 99(1):45–56.

[evz003-B26] Cingolani P, et al 2012 A program for annotating and predicting the effects of single nucleotide polymorphisms, SnpEff: SNPs in the genome of *Drosophila melanogaster* strain w1118; iso-2; iso-3. Fly 6(2):80–92. 22728672 10.4161/fly.19695 PMC3679285

[evz003-B27] Cnaani A. 2013 The tilapias’ chromosomes influencing sex determination. Cytogenet Genome Res. 141(2–3):195–205. 24107438 10.1159/000355304

[evz003-B28] Cnaani A, et al 2008 Genetics of sex determination in tilapiine species. Sex Dev. 2(1):43–54. 18418034 10.1159/000117718

[evz003-B29] Conesa A, et al 2005 Blast2GO: a universal tool for annotation, visualization and analysis in functional genomics research. Bioinformatics 21(18):3674–3676. 16081474 10.1093/bioinformatics/bti610

[evz003-B30] Conte MA, Gammerdinger WJ, Bartie KL, Penman DJ, Kocher TD. 2017 A high quality assembly of the Nile tilapia (*Oreochromis niloticus*) genome reveals the structure of two sex determination regions. BMC Genomics. 18(1):341. 28464822 10.1186/s12864-017-3723-5 PMC5414186

[evz003-B31] Danecek P, et al 2011 The variant call format and VCFtools. Bioinformatics 27(15):2156–2158. 21653522 10.1093/bioinformatics/btr330 PMC3137218

[evz003-B32] DeMarco R, Machado AA, Bisson AW, Verjovski-Almeida S. 2005 Identification of 18 new transcribed retrotransposons in *Schistosoma mansoni*. Biochem Biophys Res Commun. 333(1):230–240. 15939396 10.1016/j.bbrc.2005.05.080

[evz003-B33] Dufresnes C, Stock M, Brelsford A, Perrin N. 2014 Range-wide sex-chromosome sequence similarity supports occasional XY recombination in European tree frogs (*Hyla arborea*). PLoS One 9(6):e97959. 24892652 10.1371/journal.pone.0097959 PMC4043726

[evz003-B34] Egger B, et al 2015 Phylogeographic and phenotypic assessment of a basal haplochromine cichlid fish from Lake Chila, Zambia. Hydrobiologia 748(1):171–184.

[evz003-B35] Feulner PGD, Schwarzer J, Haesler MP, Meier JI, Seehausen O. 2018 A dense linkage lap of Lake Victoria cichlids improved the *Pundamilia* genome assembly and revealed a major QTL for sex-setermination. G3 (Bethesda) 8:2411–2420. 29760203 10.1534/g3.118.200207 PMC6027883

[evz003-B36] Fontaine A, et al 2017 Extensive genetic differentiation between homomorphic sex chromosomes in the mosquito vector, *Aedes aegypti*. Genome Biol Evol. 9(9):2322–2335. 28945882 10.1093/gbe/evx171 PMC5737474

[evz003-B37] Gammerdinger W, et al 2018a Novel sex chromosomes in 3 cichlid fishes from Lake Tanganyika. J Hered. 109(5):489–500. 29444291 10.1093/jhered/esy003

[evz003-B135] Gammerdinger WJ, Conte MA, Sandkam BA, Penman DJ, Kocher TD. 2018b Characterization of sex chromosomes in three deeply diverged species of Pseudocrinlabrinae (Teleostei: Cichlidae). Hydrobiologia Advance Access published September 26, 2018. 10.1007/s10750-018-3778-6. PMC9162429 35665074

[evz003-B38] Garg SG, Martin WF. 2016 Mitochondria, the cell cycle, and the origin of sex via a syncytial eukaryote common ancestor. Genome Biol Evol. 8(6):1950–1970. 27345956 10.1093/gbe/evw136 PMC5390555

[evz003-B39] Graves JAM. 2006 Sex chromosome specialization and degeneration in mammals. Cell 124(5):901–914. 16530039 10.1016/j.cell.2006.02.024

[evz003-B40] Graves JAM. 2008 Weird animal genomes and the evolution of vertebrate sex and sex chromosomes. Annu Rev Genet. 42(1):565–586. 18983263 10.1146/annurev.genet.42.110807.091714

[evz003-B41] Graves JAM. 2014 Avian sex, sex chromosomes, and dosage compensation in the age of genomics. Chromosome Res. 22(1):45–47. 24599719 10.1007/s10577-014-9409-9

[evz003-B42] Guerrero RF, Kirkpatrick M, Perrin N. 2012 Cryptic recombination in the ever-young sex chromosomes of Hylid frogs. J Evol Biol. 25(10):1947–1954. 22901240 10.1111/j.1420-9101.2012.02591.x

[evz003-B43] Gupta GS. 2005 Proteomics of spermatogenesis. New York: Springer.

[evz003-B44] Gurevich A, Saveliev V, Vyahhi N, Tesler G. 2013 QUAST: quality assessment tool for genome assemblies. Bioinformatics 29(8):1072–1075. 23422339 10.1093/bioinformatics/btt086 PMC3624806

[evz003-B45] Heitman J. 2015 Evolution of sexual reproduction: a view from the fungal kingdom supports an evolutionary epoch with sex before sexes. Fungal Biol Rev. 29(3–4):108–117. 26834823 10.1016/j.fbr.2015.08.002 PMC4730888

[evz003-B46] Henning F, Meyer A. 2014 The evolutionary genomics of cichlid fishes: explosive speciation and adaptation in the postgenomic era. Annu Rev Genomics Hum Genet. 15:417–414. 10.1146/annurev-genom-090413-025412 24898042

[evz003-B47] Henningsen A, Toomet O. 2016 miscTools: miscellaneous tools and utilities. Available from: https://cran.r-project.org/web/packages/miscTools/index.html; last accessed January 23, 2019.

[evz003-B48] Heule C, Salzburger W, Böhne A. 2014 Genetics of sexual development—an evolutionary playground for fish. Genetics 196(3):579–591. 24653206 10.1534/genetics.114.161158 PMC3948791

[evz003-B49] Hoff KJ, Stanke M. 2013 WebAUGUSTUS—a web service for training AUGUSTUS and predicting genes in eukaryotes. Nucleic Acids Res. 41(Web Server issue):W123–W128. 23700307 10.1093/nar/gkt418 PMC3692069

[evz003-B50] Jeffries DL, et al 2018 A rapid rate of sex-chromosome turnover and non-random transitions in true frogs. Nat Commun. 9(1):4088. 30291233 10.1038/s41467-018-06517-2 PMC6173717

[evz003-B51] Kamiya T, et al 2012 A trans-species missense SNP in *amhr2* is associated with sex determination in the tiger pufferfish, *Takifugu rubripes* (Fugu). PLoS Genet. 8(7):e1002798. 22807687 10.1371/journal.pgen.1002798 PMC3395601

[evz003-B52] Katoh K, Rozewicki J, Yamada KD. 2017. MAFFT online service: multiple sequence alignment, interactive sequence choice and visualization. Brief Bioinformatics. bbx108. 10.1093/bib/bbx108 PMC6781576 28968734

[evz003-B53] Katongo C, Koblmüller S, Duftner N, Makasa L, Sturmbauer C. 2005 Phylogeography and speciation in the *Pseudocrenilabrus philander* species complex in Zambian Rivers. Hydrobiologia 542(1):221–233.

[evz003-B54] Kiełbasa SM, Wan R, Sato K, Horton P, Frith MC. 2011 Adaptive seeds tame genomic sequence comparison. Genome Res. 21(3):487–493. 21209072 10.1101/gr.113985.110 PMC3044862

[evz003-B55] Kitano J, Peichel C. 2012 Turnover of sex chromosomes and speciation in fishes. Environ Biol Fishes 94(3):549–558. 26069393 10.1007/s10641-011-9853-8 PMC4459657

[evz003-B56] Kiuchi T, et al 2014 A single female-specific piRNA is the primary determiner of sex in the silkworm. Nature 509(7502):633–636. 24828047 10.1038/nature13315

[evz003-B57] Koblmuller S, Katongo C, Phiri H, Sturmbauer C. 2012 Past connection of the upper reaches of a Lake Tanganyika tributary with the upper Congo drainage suggested by genetic data of riverine cichlid fishes. Afr Zool. 47:182–186.

[evz003-B58] Kondo M, et al 2006 Genomic organization of the sex-determining and adjacent regions of the sex chromosomes of medaka. Genome Res. 16(7):815–826. 16751340 10.1101/gr.5016106 PMC1484449

[evz003-B59] Kornfield I, Smith P. 2000 African cichlid fishes: model system for evolutionary biology. Annu Rev Ecol Syst. 31(1):163–196.

[evz003-B60] Kudo Y, et al 2015 A microsatellite-based genetic linkage map and putative sex-determining genomic regions in Lake Victoria cichlids. Gene 560(2):156–164. 25639358 10.1016/j.gene.2015.01.057

[evz003-B61] Lahn BT, Page DC. 1999 Four evolutionary strata on the human X chromosome. Science 286(5441):964–967. 10542153 10.1126/science.286.5441.964

[evz003-B62] Lee BY, Hulata G, Kocher TD. 2004 Two unlinked loci controlling the sex of blue tilapia (*Oreochromis aureus*). Heredity 92(6):543–549. 15100706 10.1038/sj.hdy.6800453

[evz003-B63] Li D, Liu C-M, Luo R, Sadakane K, Lam T-W. 2015 MEGAHIT: an ultra-fast single-node solution for large and complex metagenomics assembly via succinct de Bruijn graph. Bioinformatics 31(10):1674–1676. 25609793 10.1093/bioinformatics/btv033

[evz003-B64] Li H, Durbin R. 2009 Fast and accurate short read alignment with Burrows–Wheeler transform. Bioinformatics 25(14):1754–1760. 19451168 10.1093/bioinformatics/btp324 PMC2705234

[evz003-B65] Li H, et al 2009 The sequence alignment/map format and SAMtools. Bioinformatics 25(16):2078–2079. 19505943 10.1093/bioinformatics/btp352 PMC2723002

[evz003-B66] Lode T. 2012 Oviparity or viviparity? That is the question … Reprod Biol. 12:259–264. 23153695 10.1016/j.repbio.2012.09.001

[evz003-B67] Magoc T, Salzberg SL. 2011 FLASH: fast length adjustment of short reads to improve genome assemblies. Bioinformatics 27(21):2957–2963. 21903629 10.1093/bioinformatics/btr507 PMC3198573

[evz003-B68] Malmstrøm M, Matschiner M, Tørresen OK, Jakobsen KS, Jentoft S. 2017 Whole genome sequencing data and de novo draft assemblies for 66 teleost species. Sci Data 4:160132. 28094797 10.1038/sdata.2016.132 PMC5240625

[evz003-B69] Marshall Graves JA, Peichel CL. 2010 Are homologies in vertebrate sex determination due to shared ancestry or to limited options? Genome Biol. 11(4):205. 20441602 10.1186/gb-2010-11-4-205 PMC2884537

[evz003-B70] Martin SH, Van Belleghem SM. 2017 Exploring evolutionary relationships across the genome using topology weighting. Genetics 206(1):429–438. 28341652 10.1534/genetics.116.194720 PMC5419486

[evz003-B71] McKenna A, et al 2010 The Genome Analysis Toolkit: a MapReduce framework for analyzing next-generation DNA sequencing data. Genome Res. 20(9):1297–1303. 20644199 10.1101/gr.107524.110 PMC2928508

[evz003-B136] Milansky et al. 2018 Whole-genome sequences of Malawi cichlids reveal multiple radiations interconnected by gene flow. Nat Ecol Evol. 2(12):1940–1955. 30455444 10.1038/s41559-018-0717-x PMC6443041

[evz003-B72] Meynert AM, Ansari M, FitzPatrick DR, Taylor MS. 2014 Variant detection sensitivity and biases in whole genome and exome sequencing. BMC Bioinformatics 15:247. 25038816 10.1186/1471-2105-15-247 PMC4122774

[evz003-B73] Miura I. 2007 An evolutionary witness: the frog *Rana rugosa* underwent change of heterogametic sex from XY male to ZW female. Sex Dev. 1(6):323–331. 18391544 10.1159/000111764

[evz003-B74] Miyagi R, Terai Y. 2013 The diversity of male nuptial coloration leads to species diversity in Lake Victoria cichlids. Genes Genet Syst. 88(3):145–153. 24025243 10.1266/ggs.88.145

[evz003-B75] Moore EC, Roberts RB. 2013 Polygenic sex determination. Curr Biol. 23(12):R510–R512. 23787041 10.1016/j.cub.2013.04.004

[evz003-B76] Muller HJ. 1918 Genetic variability, twin hybrids and constant hybrids in a case of balanced lethal factors. Genetics 3(5):422–499. 17245914 10.1093/genetics/3.5.422 PMC1200446

[evz003-B77] Muller HJ. 1932 Some genetic aspects of sex. Am Nat. 66(703):118–138.

[evz003-B78] Muschick M, Indermaur A, Salzburger W. 2012 Convergent evolution within an adaptive radiation of cichlid fishes. Curr Biol. 22(24):2362–2368. 23159601 10.1016/j.cub.2012.10.048

[evz003-B79] Myers EW, et al 2000 A whole-genome assembly of *Drosophila*. Science 287(5461):2196–2204. 10731133 10.1126/science.287.5461.2196

[evz003-B80] Myosho T, et al 2012 Tracing the emergence of a novel sex-determining gene in medaka, *Oryzias luzonensis*. Genetics 191(1):163–170. 22367037 10.1534/genetics.111.137497 PMC3338257

[evz003-B81] Nei M, Li WH. 1979 Mathematical model for studying genetic variation in terms of restriction endonucleases. Proc Natl Acad Sci U S A. 76(10):5269–5273. 291943 10.1073/pnas.76.10.5269 PMC413122

[evz003-B82] O’Quin CT. 2014 The genetic basis of pigment pattern differentiation in Lake Malawi African cichlids. College Park (MD): University of Maryland.

[evz003-B83] Ottolenghi C, et al 2005 Foxl2 is required for commitment to ovary differentiation. Hum Mol Genet. 14(14):2053–2062. 15944199 10.1093/hmg/ddi210

[evz003-B84] Pannell JR. 2017 Plant sex determination. Curr Biol. 27(5):R191–R197. 28267976 10.1016/j.cub.2017.01.052

[evz003-B85] Parnell NF, Hulsey CD, Streelman JT. 2012 The genetic basis of a complex functional system. Evolution 66(11):3352–3366. 23106702 10.1111/j.1558-5646.2012.01688.x PMC3490443

[evz003-B86] Parnell NF, Streelman JT. 2013 Genetic interactions controlling sex and color establish the potential for sexual conflict in Lake Malawi cichlid fishes. Heredity 110(3):239–246. 23092997 10.1038/hdy.2012.73 PMC3668650

[evz003-B87] Patterson N, Price AL, Reich D. 2006 Population structure and eigenanalysis. PLoS Genet. 2:2074–2093. 10.1371/journal.pgen.0020190 PMC1713260 17194218

[evz003-B88] Perrin N. 2016 Random sex determination: when developmental noise tips the sex balance. Bioessays 38(12):1218–1226. 27641730 10.1002/bies.201600093

[evz003-B89] Peterson EN, Cline ME, Moore EC, Roberts NB, Roberts RB. 2017 Genetic sex determination in *Astatotilapia calliptera*, a prototype species for the Lake Malawi cichlid radiation. Naturwissenschaften 104(5–6):41. 28444435 10.1007/s00114-017-1462-8

[evz003-B90] Posada D. 2008 jModelTest: phylogenetic model averaging. Mol Biol Evol. 25(7):1253–1256. 18397919 10.1093/molbev/msn083

[evz003-B91] R Core Team. 2017 R: A language and environment for statistical computing. Vienna (Austria): R Foundation for Statistical Computing. Available from: https://www.r-project.org/; last accessed January 23, 2019.

[evz003-B92] Reddon AR, Hurd PL. 2013 Water pH during early development influences sex ratio and male morph in a West African cichlid fish, *Pelvicachromis pulcher*. Zoology (Jena) 116(3):139–143. 23474178 10.1016/j.zool.2012.11.001

[evz003-B93] Rice P, Longden I, Bleasby A. 2000 EMBOSS: the European molecular biology open software suite. Trends Genet. 16(6):276–277. 10827456 10.1016/s0168-9525(00)02024-2

[evz003-B94] Roberts NB, et al 2016 Polygenic sex determination in the cichlid fish *Astatotilapia burtoni*. BMC Genomics. 17(1):835. 27784286 10.1186/s12864-016-3177-1 PMC5080751

[evz003-B95] Roberts RB, Ser JR, Kocher TD. 2009 Sexual conflict resolved by invasion of a novel sex determiner in Lake Malawi cichlid fishes. Science 326(5955):998–1001. 19797625 10.1126/science.1174705 PMC3174268

[evz003-B96] Rodrigues N, Dufresnes C. 2017 Using conventional F-statistics to study unconventional sex-chromosome differentiation. PeerJ 5:e3207. 28462023 10.7717/peerj.3207 PMC5410149

[evz003-B97] Römer U, Beisenherz W. 1996 Environmental determination of sex in Apistogrammai (Cichlidae) and two other freshwater fishes (Teleostei). J Fish Biol. 48:714–725.

[evz003-B98] Rondeau E, et al 2013 Genomics of sablefish (*Anoplopoma fimbria*): expressed genes, mitochondrial phylogeny, linkage map and identification of a putative sex gene. BMC Genomics. 14:452. 23829495 10.1186/1471-2164-14-452 PMC3708741

[evz003-B99] Ronquist F, et al 2012 MrBayes 3.2: efficient Bayesian phylogenetic inference and model choice across a large model space. Syst Biol. 61(3):539–542. 22357727 10.1093/sysbio/sys029 PMC3329765

[evz003-B100] Salzburger W. 2018 Understanding explosive diversification through cichlid fish genomics. Nat Rev Genet. 19(11):705–717. 30111830 10.1038/s41576-018-0043-9

[evz003-B101] Salzburger W, Mack T, Verheyen E, Meyer A. 2005 Out of Tanganyika: genesis, explosive speciation, key-innovations and phylogeography of the haplochromine cichlid fishes. BMC Evol Biol. 5:17. 15723698 10.1186/1471-2148-5-17 PMC554777

[evz003-B102] Salzburger W, Meyer A. 2004 The species flocks of East African cichlid fishes: recent advances in molecular phylogenetics and population genetics. Naturwissenschaften 91(6):277–290. 15241604 10.1007/s00114-004-0528-6

[evz003-B103] Schartl M, Schmid M, Nanda I. 2016 Dynamics of vertebrate sex chromosome evolution: from equal size to giants and dwarfs. Chromosoma 125(3):553–571. 26715206 10.1007/s00412-015-0569-y

[evz003-B104] Schwarzer J, Misof B, Tautz D, Schliewen UK. 2009 The root of the East African cichlid radiations. BMC Evol Biol. 9:186. 19656365 10.1186/1471-2148-9-186 PMC2739198

[evz003-B105] Schwarzer J, et al 2012 Repeated trans-watershed hybridization among haplochromine cichlids (Cichlidae) was triggered by Neogene landscape evolution. Proc Biol Sci. 279(1746):4389–4398. 22951733 10.1098/rspb.2012.1667 PMC3479809

[evz003-B106] Seehausen O. 2015 Process and pattern in cichlid radiations—inferences for understanding unusually high rates of evolutionary diversification. New Phytol. 207(2):304–312. 25983053 10.1111/nph.13450

[evz003-B107] Sefc KM. 2011 Mating and parental care in Lake Tanganyika’s cichlids. Int J Evol Biol. 2011:470875. 21822482 10.4061/2011/470875 PMC3142683

[evz003-B108] Ser JR, Roberts RB, Kocher TD. 2010 Multiple interacting loci control sex determination in Lake Malawi cichlid fish. Evolution 64(2):486–501. 19863587 10.1111/j.1558-5646.2009.00871.x PMC3176681

[evz003-B109] Sessions SK, Bizjak Mali L, Green DM, Trifonov V, Ferguson-Smith M. 2016 Evidence for sex chromosome turnover in proteid salamanders. Cytogenet Genome Res. 148(4):305–313. 27351721 10.1159/000446882

[evz003-B110] Simao FA, Waterhouse RM, Ioannidis P, Kriventseva EV, Zdobnov EM. 2015 BUSCO: assessing genome assembly and annotation completeness with single-copy orthologs. Bioinformatics 31(19):3210–3212. 26059717 10.1093/bioinformatics/btv351

[evz003-B111] Sinclair AH, et al 1990 A gene from the human sex-determining region encodes a protein with homology to a conserved DNA-binding motif. Nature 346(6281):240–244. 1695712 10.1038/346240a0

[evz003-B112] Speijer D, Lukes J, Elias M. 2015 Sex is a ubiquitous, ancient, and inherent attribute of eukaryotic life. Proc Natl Acad Sci U S A. 112(29):8827–8834. 26195746 10.1073/pnas.1501725112 PMC4517231

[evz003-B113] Stamatakis A. 2014 RAxML version 8: a tool for phylogenetic analysis and post-analysis of large phylogenies. Bioinformatics 30(9):1312–1313. 24451623 10.1093/bioinformatics/btu033 PMC3998144

[evz003-B114] Stock M, et al 2011 Ever-young sex chromosomes in European tree frogs. PLoS Biol. 9:e1001062. 21629756 10.1371/journal.pbio.1001062 PMC3100596

[evz003-B115] Sturtevant AH. 1921 A case of rearrangement of genes in *Drosophila*. Proc Natl Acad Sci U S A. 7(8):235–237. 16576597 10.1073/pnas.7.8.235 PMC1084859

[evz003-B116] Tomaszkiewicz M, Medvedev P, Makova KD. 2017 Y and W chromosome assemblies: approaches and discoveries. Trends Genet. 33(4):266–282. 28236503 10.1016/j.tig.2017.01.008

[evz003-B117] Turner GF, Seehausen O, Knight ME, Allender CJ, Robinson RL. 2001 How many species of cichlid fishes are there in African lakes? Mol Ecol. 10(3):793–806. 11298988 10.1046/j.1365-294x.2001.01200.x

[evz003-B118] Uhlenhaut NH, Treier M. 2011 Forkhead transcription factors in ovarian function. Reproduction 142(4):489–495. 21810859 10.1530/REP-11-0092

[evz003-B119] van Doorn GS, Kirkpatrick M. 2007 Turnover of sex chromosomes induced by sexual conflict. Nature 449(7164):909–912. 17943130 10.1038/nature06178

[evz003-B120] van Doorn GS, Kirkpatrick M. 2010 Transitions between male and female heterogamety caused by sex-antagonistic selection. Genetics 186(2):629–645. 20628036 10.1534/genetics.110.118596 PMC2954476

[evz003-B121] Verheyen E, Salzburger W, Snoeks J, Meyer A. 2003 Origin of the superflock of cichlid fishes from Lake Victoria, East Africa. Science 300(5617):325–329. 12649486 10.1126/science.1080699

[evz003-B122] Vicoso B, Emerson JJ, Zektser Y, Mahajan S, Bachtrog D. 2013 Comparative sex chromosome genomics in snakes: differentiation, evolutionary strata, and lack of global dosage compensation. PLoS Biol 11(8):e1001643. 10.1371/journal.pbio.1001643 PMC3754893 24015111

[evz003-B123] Villesen P. 2007 FaBox: an online toolbox for FASTA sequences. Mol Ecol Notes 7(6):965–968.

[evz003-B124] Volff J-N, Nanda I, Schmid M, Schartl M. 2007 Governing sex determination in fish: regulatory putsches and ephemeral dictators. Sex Dev. 1(2):85–99. 18391519 10.1159/000100030

[evz003-B125] Westergaard M. 1958 The mechanism of sex determination in dioecious flowering plants. Adv Genet. 9:217–281. 13520443 10.1016/s0065-2660(08)60163-7

[evz003-B126] Wickham H. 2007 Reshaping data with the reshape package. J Stat Softw. 21(12):1–20.

[evz003-B127] Wickham H. 2009 ggplot2: elegant graphics for data analysis. New York: Springer Verlag.

[evz003-B128] Wilhelm D, Englert C. 2002 The Wilms tumor suppressor WT1 regulates early gonad development by activation of Sf1. Genes Dev. 16(14):1839–1851. 12130543 10.1101/gad.220102 PMC186395

[evz003-B129] Xiao J, et al 2015 Transcriptome analysis revealed positive selection of immune-related genes in tilapia. Fish Shellfish Immunol. 44(1):60–65. 25659230 10.1016/j.fsi.2015.01.022

[evz003-B130] Yan W, Ma L, Burns KH, Matzuk MM. 2004 Haploinsufficiency of kelch-like protein homolog 10 causes infertility in male mice. Proc Natl Acad Sci U S A. 101(20):7793–7798. 15136734 10.1073/pnas.0308025101 PMC419685

[evz003-B131] Yang JA, et al 2010 Common SNPs explain a large proportion of the heritability for human height. Nat Genet. 42(7):565–569. 20562875 10.1038/ng.608 PMC3232052

[evz003-B132] Yoshida K, et al 2011 B chromosomes have a functional effect on female sex determination in Lake Victoria cichlid fishes. PLoS Genet. 7(8):e1002203. 21876673 10.1371/journal.pgen.1002203 PMC3158035

[evz003-B133] Yoshida K, et al 2014 Sex chromosome turnover contributes to genomic divergence between incipient stickleback species. PLoS Genet. 10(3):e1004223. 24625862 10.1371/journal.pgen.1004223 PMC3953013

[evz003-B134] Zeileis A, Grothendieck G. 2005 zoo: S3 infrastructure for regular and irregular time series. J Stat Softw. 14:1–27.

