# Supplementary material for: Repeated Evolution Versus Common Ancestry: Sex Chromosome Evolution in the Haplochromine Cichlid Pseudocrenilabrus philander
Source: Genome Biol Evol. 2019 Jan 12;11(2):439–58. doi: 10.1093/gbe/evz003 (PMC6375353; doi:10.1093/gbe/evz003)
Supplement: Supplementary Data [file evz003_supp.zip › SupplementaryMaterialFiguresS1_S17.pdf]

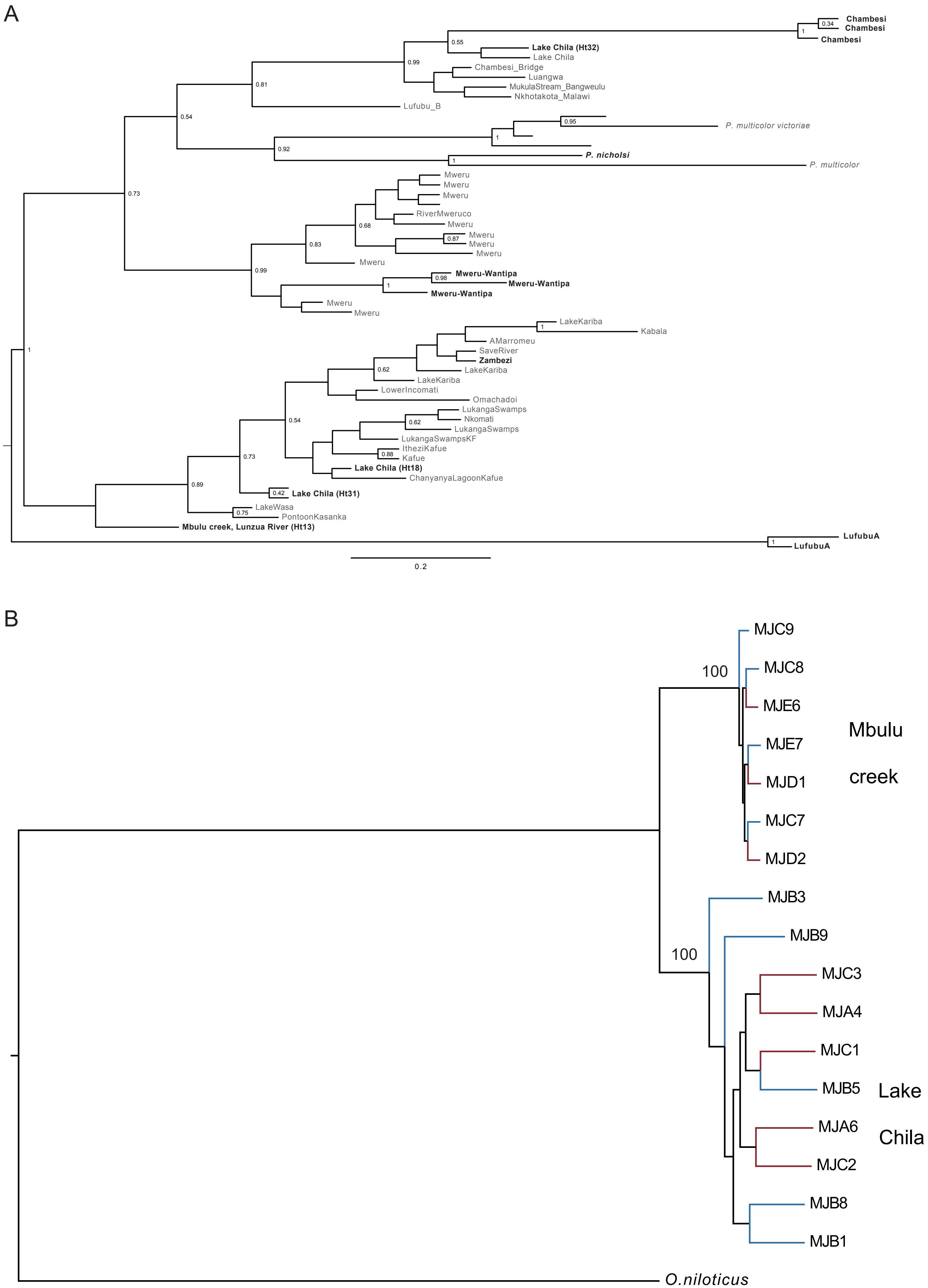
A
Chambesi
Chambesi
Chambesi
0.34
1
0.55
Lake Chila (Ht32)
Lake Chila
0.99
Chambesi_Bridge
Luangwa
MukulaStream_Bangweulu
Nkhotakota_Malawi
0.81
Lufubu_B
0.54
0.95
P. multicolor victoriae
1
0.92
P. nicholsi
1
P. multicolor
Mweru
Mweru
Mweru
0.73
RiverMweruco
0.68
Mweru
Mweru
0.87
Mweru
Mweru
0.83
Mweru
0.99
Mweru-Wantipa
0.98
Mweru-Wantipa
1
Mweru-Wantipa
Mweru
Mweru
LakeKariba
1
Kabala
AMarromeu
SaveRiver
Zambezi
1
0.62
LakeKariba
LakeKariba
LowerIncomati
Omachadoi
LukangaSwamps
0.54
0.62
Nkomati
LukangaSwamps
LukangaSwampsKF
ItheziKafue
0.88
Kafue
0.73
Lake Chila (Ht18)
ChanyanyaLagoonKafue
0.89
0.42
Lake Chila (Ht31)
LakeWasa
0.75
PontoonKasanka
Mbulu creek, Lunzua River (Ht13)
LufubuA
1
LufubuA
0.2
B
MJC9
100
MJC8
MJE6
MJE7
Mbulu
MJD1
creek
MJC7
MJD2
MJB3
MJB9
100
MJC3
MJA4
MJC1
MJB5
Lake
MJA6
Chila
MJC2
MJB8
MJB1
O.niloticus

Figure S1

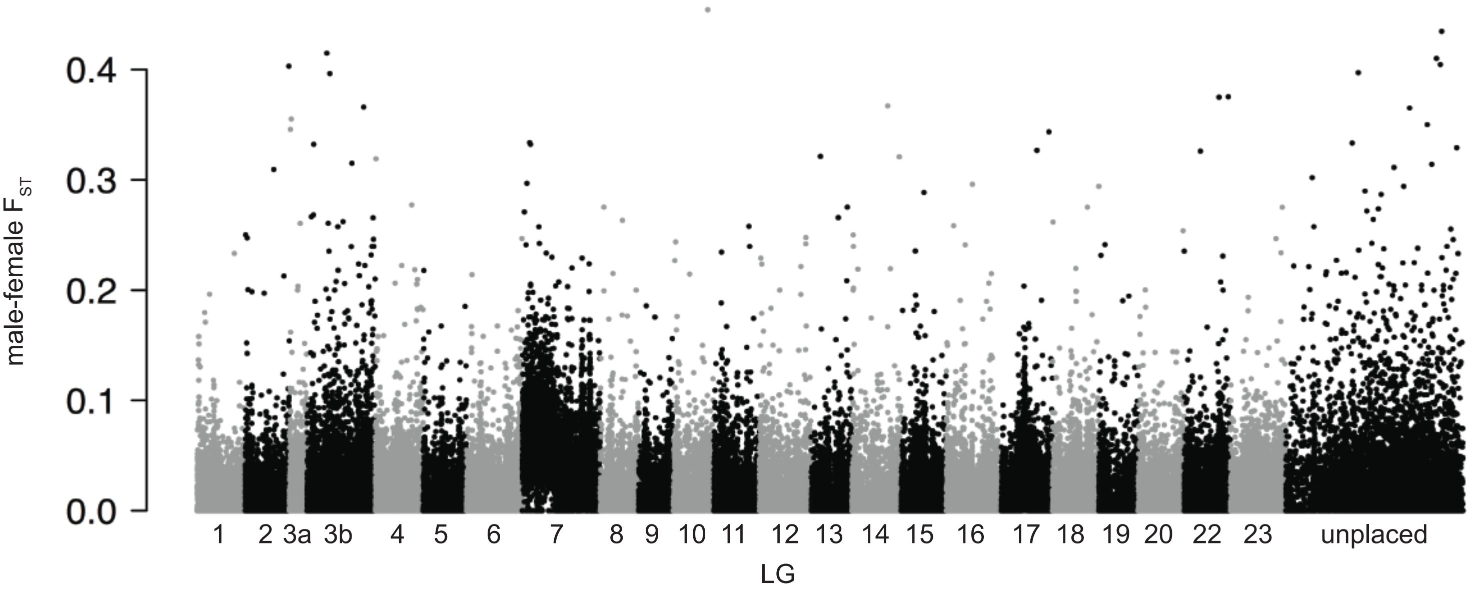
0.4
0.3
0.2
0.1
0.0
male-female F_{ST}
1 2 3a 3b 4 5 6 7 8 9 10 11 12 13 14 15 16 17 18 19 20 22 23 unplaced
LG

Figure S2

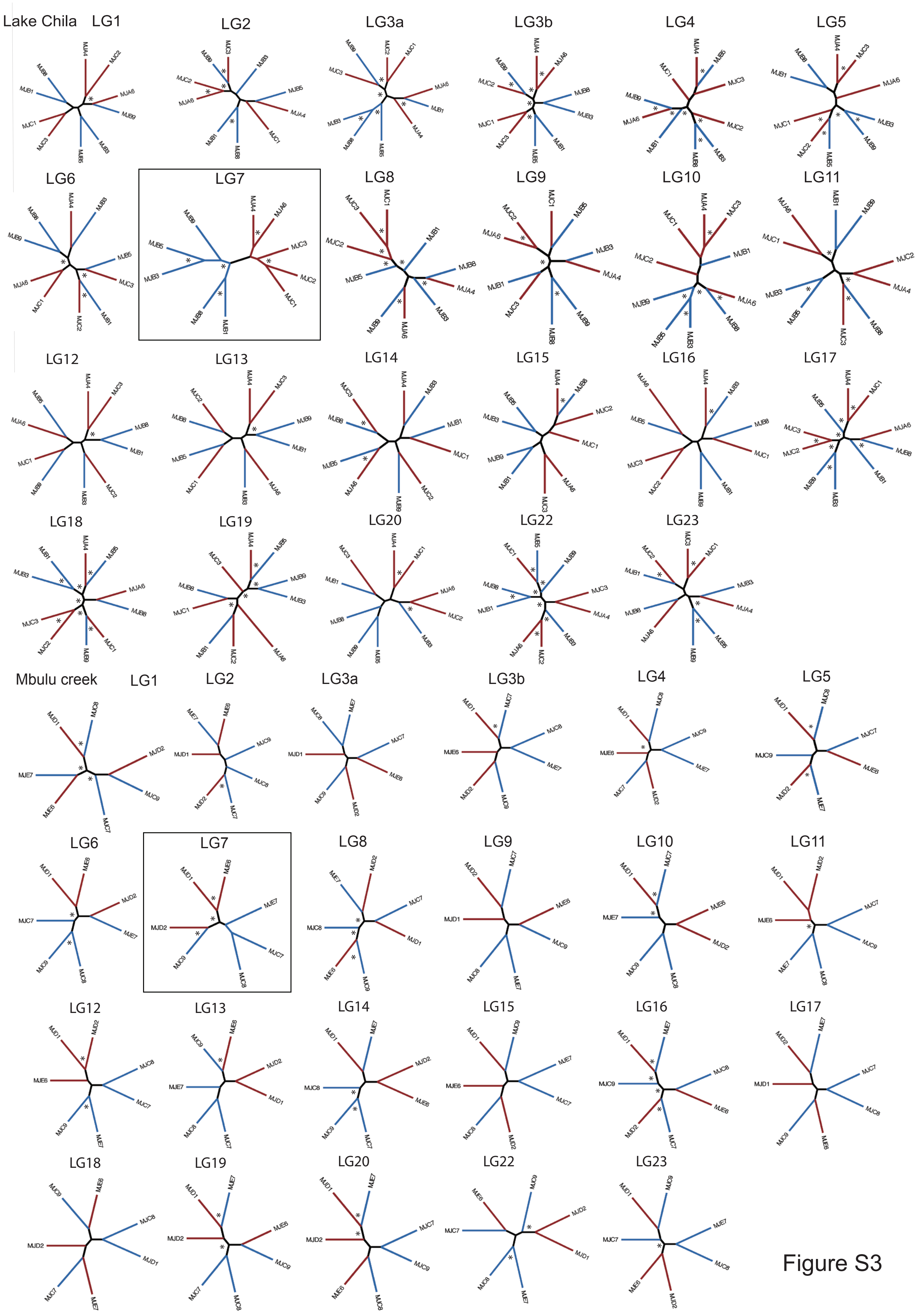
Lake Chila
LG1
LG2
LG3a
LG3b
LG4
LG5
LG6
LG7
LG8
LG9
LG10
LG11
LG12
LG13
LG14
LG15
LG16
LG17
LG18
LG19
LG20
LG22
LG23
Mbulu creek
LG1
LG2
LG3a
LG3b
LG4
LG5
LG6
LG7
LG8
LG9
LG10
LG11
LG12
LG13
LG14
LG15
LG16
LG17
LG18
LG19
LG20
LG22
LG23

Figure S3

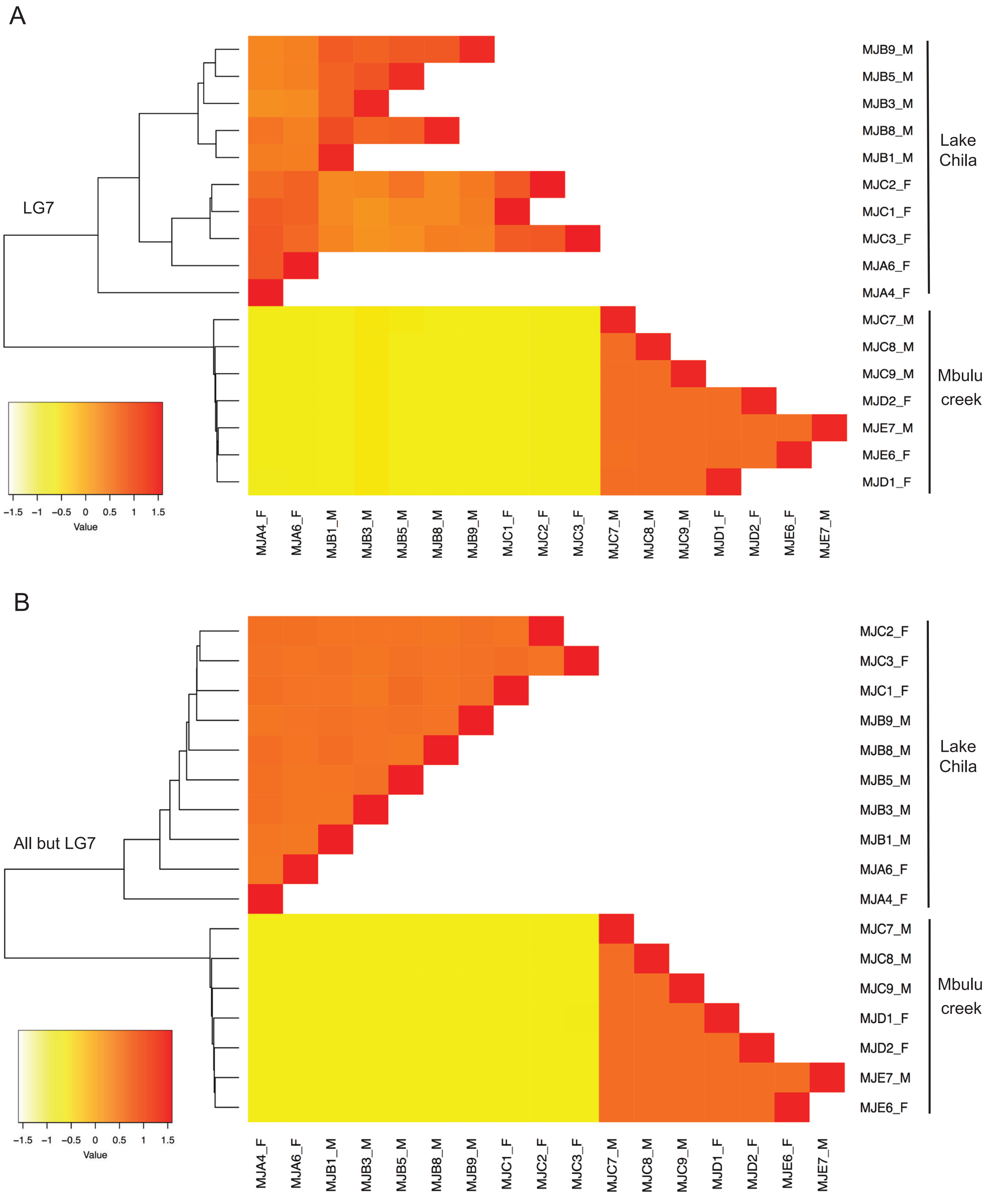
A
LG7
MJB9_M
MJB5_M
MJB3_M
MJB8_M
MJB1_M
MJC2_F
MJC1_F
MJC3_F
MJA6_F
MJA4_F
MJC7_M
MJC8_M
MJC9_M
MJD2_F
MJE7_M
MJE6_F
MJD1_F
Lake Chila
Mbulu creek
−1.5 −1 −0.5 0 0.5 1 1.5
Value
MJA4_F MJA6_F MJB1_M MJB3_M MJB5_M MJB8_M MJB9_M MJC1_F MJC2_F MJC3_F MJC7_M MJC8_M MJC9_M MJD1_F MJD2_F MJE6_F MJE7_M
B
All but LG7
MJC2_F
MJC3_F
MJC1_F
MJB9_M
MJB8_M
MJB5_M
MJB3_M
MJB1_M
MJA6_F
MJA4_F
MJC7_M
MJC8_M
MJC9_M
MJD1_F
MJD2_F
MJE7_M
MJE6_F
Lake Chila
Mbulu creek
−1.5 −1 −0.5 0 0.5 1 1.5
Value
MJA4_F MJA6_F MJB1_M MJB3_M MJB5_M MJB8_M MJB9_M MJC1_F MJC2_F MJC3_F MJC7_M MJC8_M MJC9_M MJD1_F MJD2_F MJE6_F MJE7_M

Figure S4

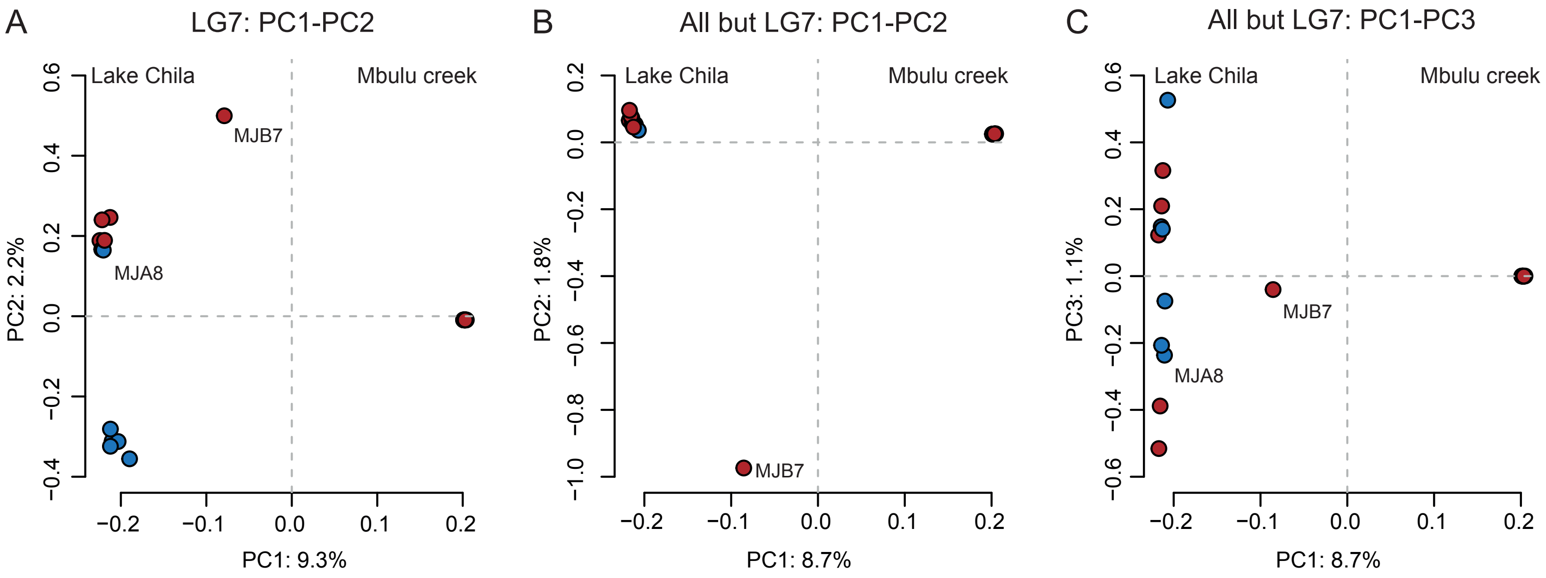
A
LG7: PC1-PC2
Lake Chila
Mbulu creek
MJB7
MJA8
PC2: 2.2%
PC1: 9.3%
B
All but LG7: PC1-PC2
Lake Chila
Mbulu creek
MJB7
PC2: 1.8%
PC1: 8.7%
C
All but LG7: PC1-PC3
Lake Chila
Mbulu creek
MJB7
MJA8
PC3: 1.1%
PC1: 8.7%

Figure S5

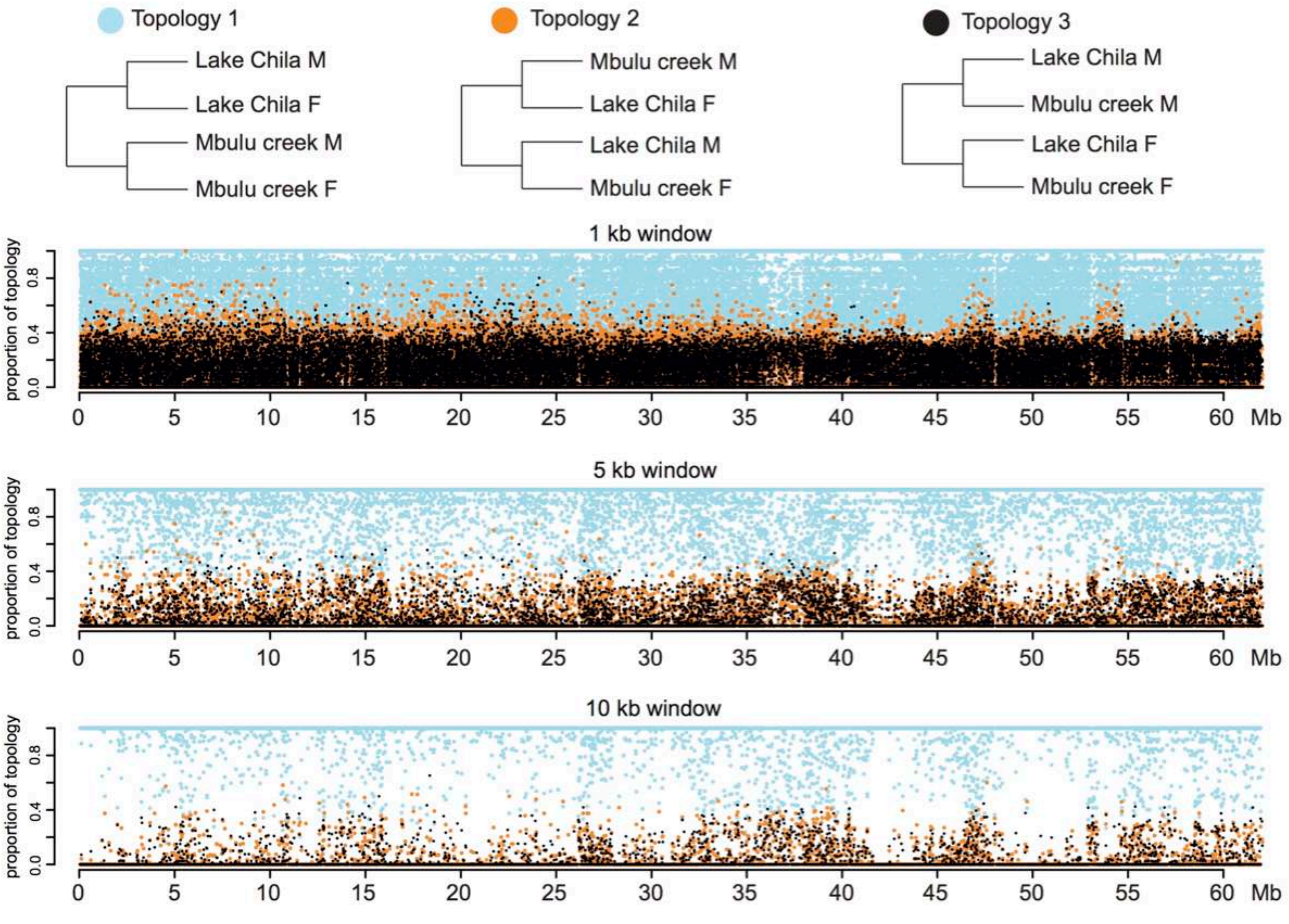

Topology 1
Lake Chila M
Lake Chila F
Mbulu creek M
Mbulu creek F
Topology 2
Mbulu creek M
Lake Chila F
Lake Chila M
Mbulu creek F
Topology 3
Lake Chila M
Mbulu creek M
Lake Chila F
Mbulu creek F
1 kb window
5 kb window
10 kb window
proportion of topology
0.0
0.4
0.8
0
5
10
15
20
25
30
35
40
45
50
55
60
Mb

Figure S6

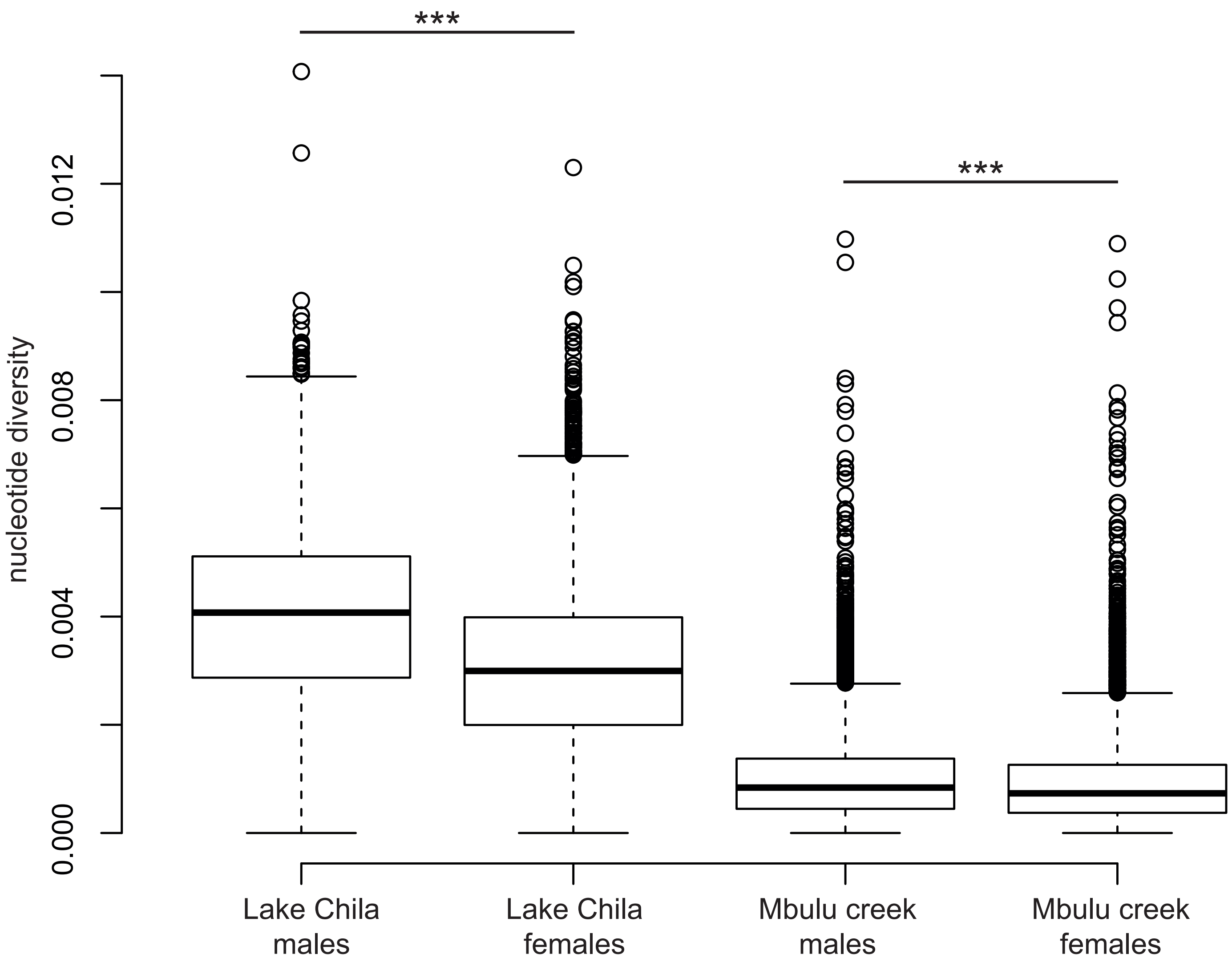
***
***
0.000
0.004
0.008
0.012
nucleotide diversity
Lake Chila males
Lake Chila females
Mbulu creek males
Mbulu creek females

Figure S7

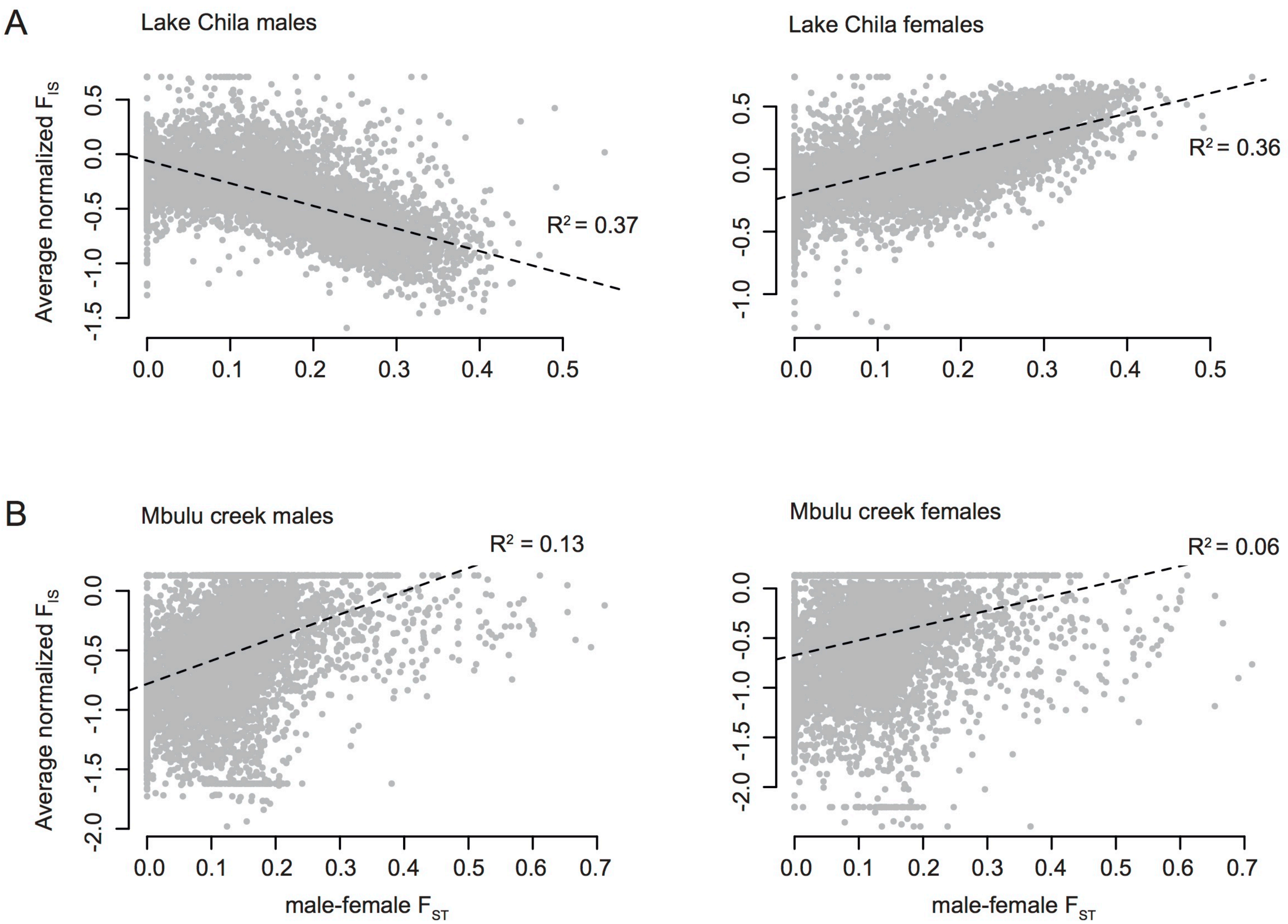
A
Lake Chila males
Lake Chila females
$R^2 = 0.37$
$R^2 = 0.36$
B
Mbulu creek males
Mbulu creek females
$R^2 = 0.13$
$R^2 = 0.06$
Average normalized F_{IS}
male-female F_{ST}

Figure S8

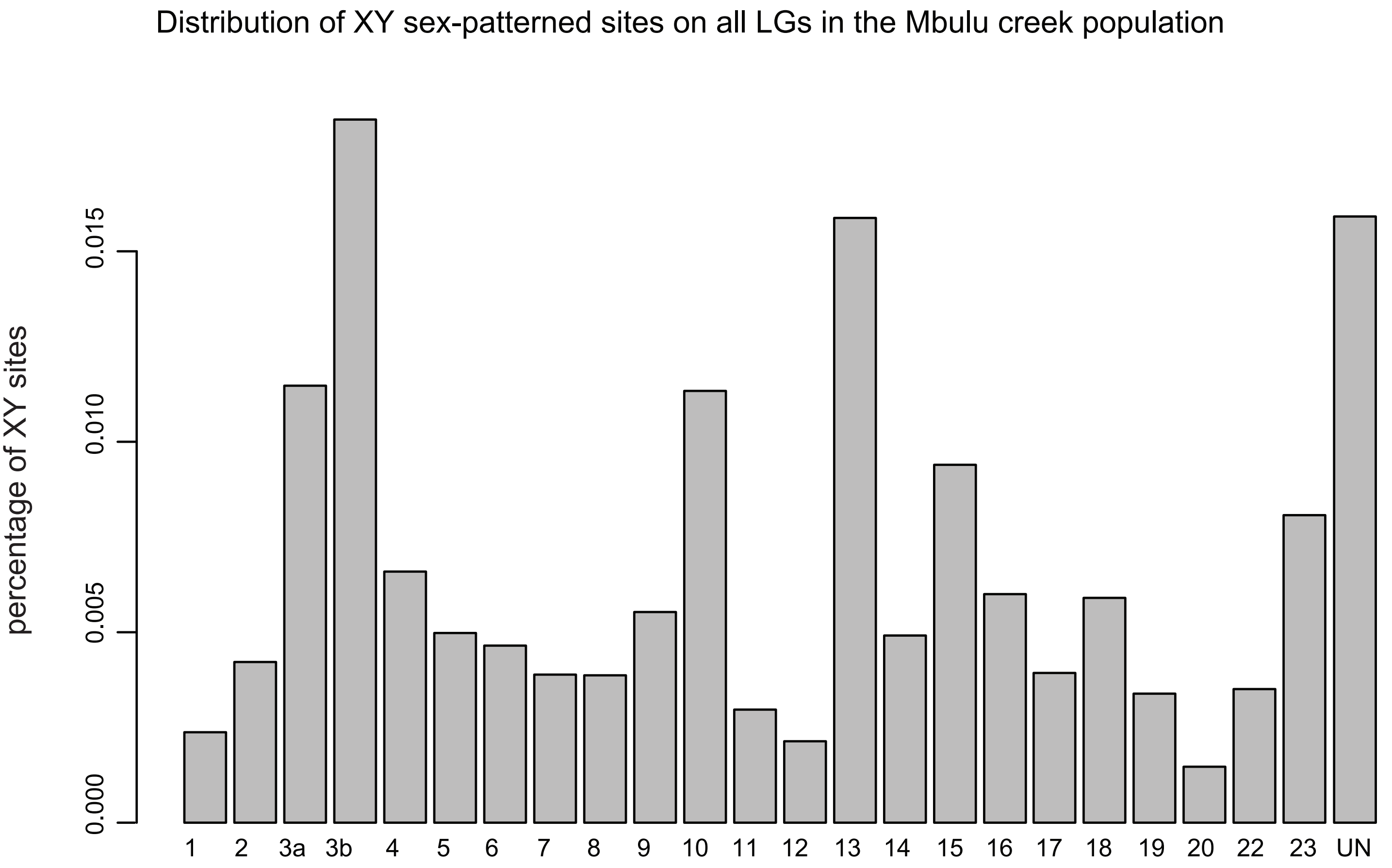
Distribution of XY sex-patterned sites on all LGs in the Mbulu creek population
percentage of XY sites
0.000
0.005
0.010
0.015
1
2
3a
3b
4
5
6
7
8
9
10
11
12
13
14
15
16
17
18
19
20
22
23
UN

Figure S9

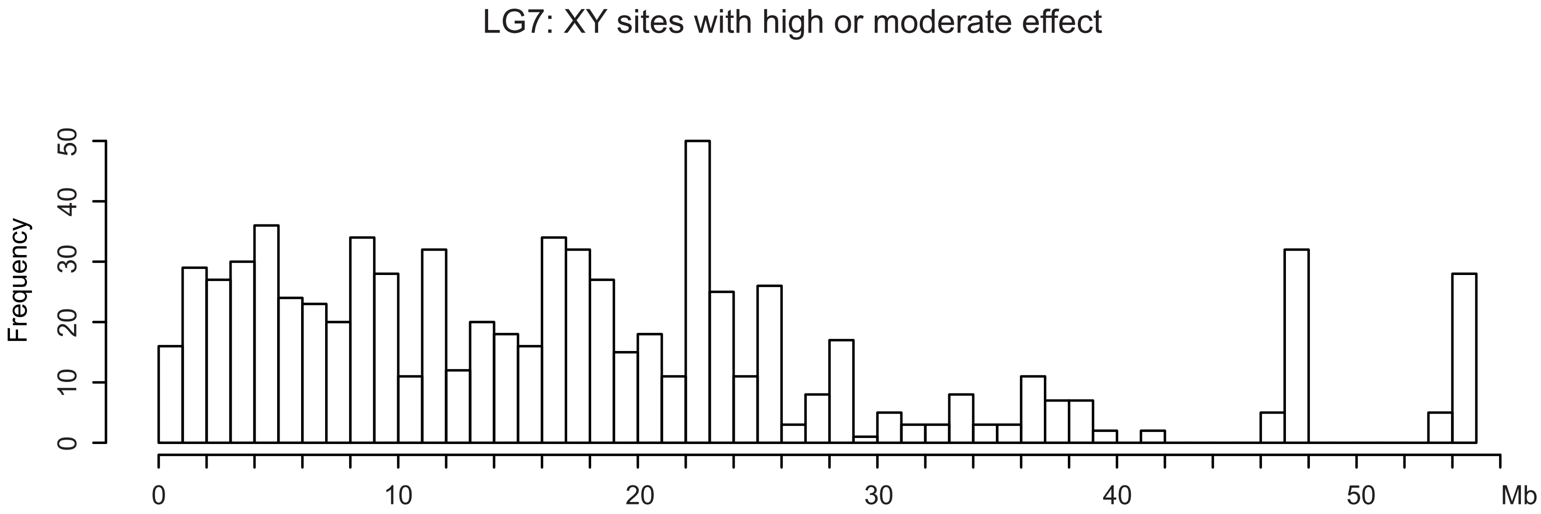
LG7: XY sites with high or moderate effect
Frequency
0
10
20
30
40
50
0
10
20
30
40
50
Mb

Figure S10

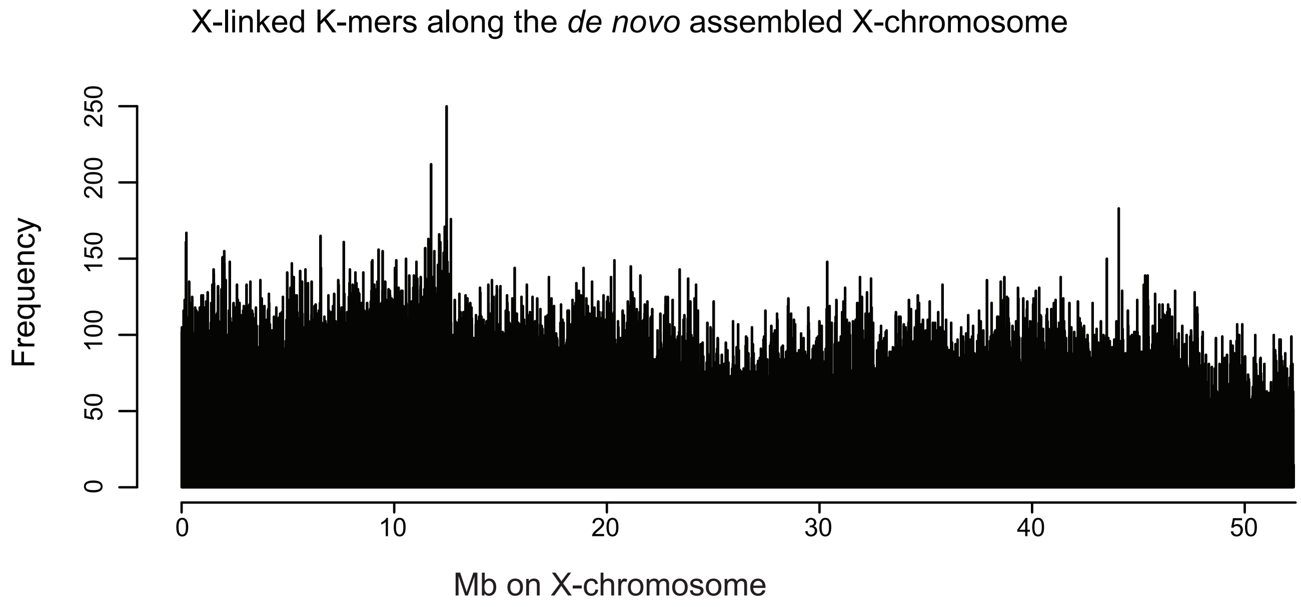

X-linked K-mers along the de novo assembled X-chromosome
Frequency
0
50
100
150
200
250
0
10
20
30
40
50
Mb on X-chromosome

Figure S11

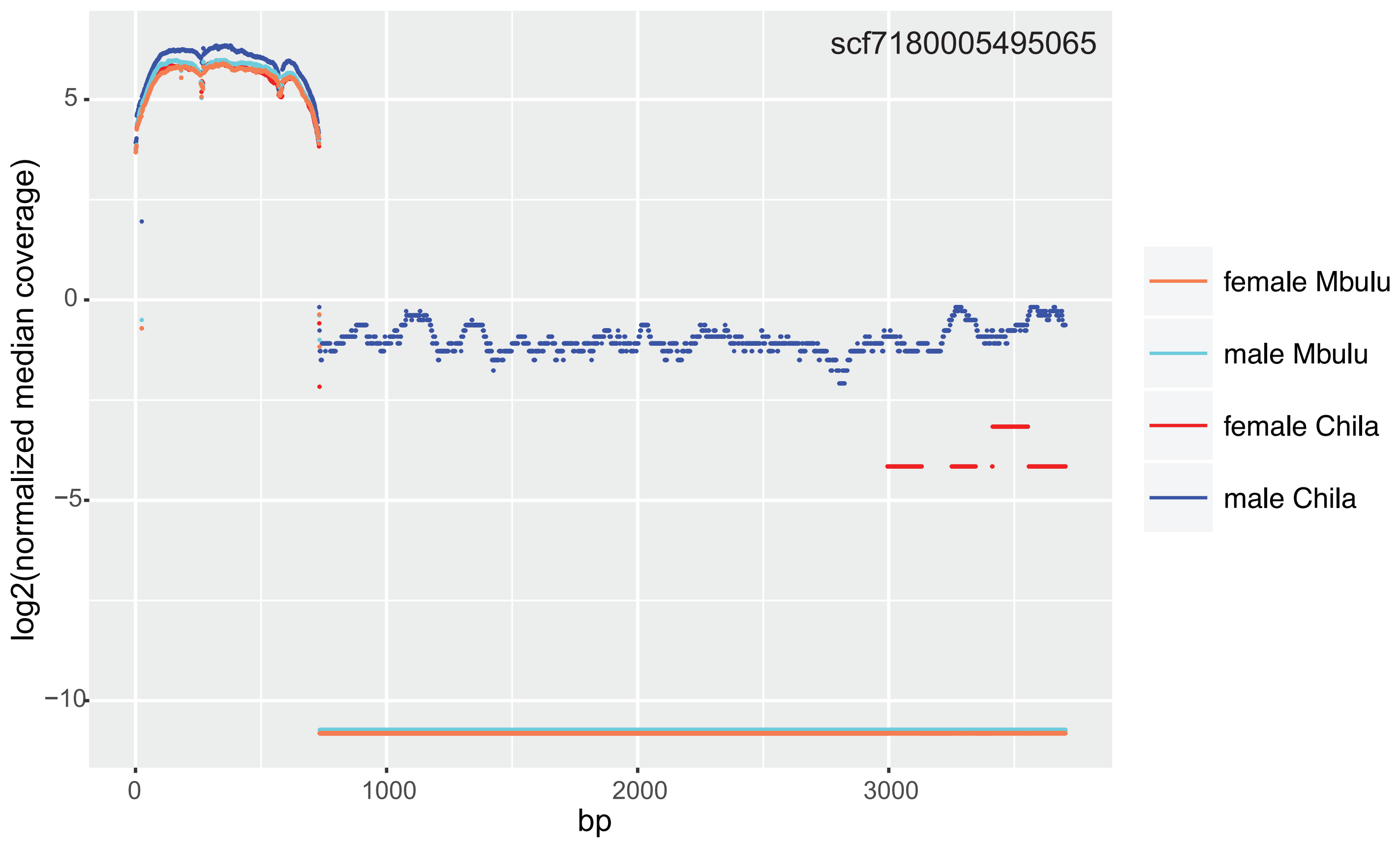
scf7180005495065
log2(normalized median coverage)
5
0
−5
−10
0
1000
2000
3000
bp
female Mbulu
male Mbulu
female Chila
male Chila

Figure S12

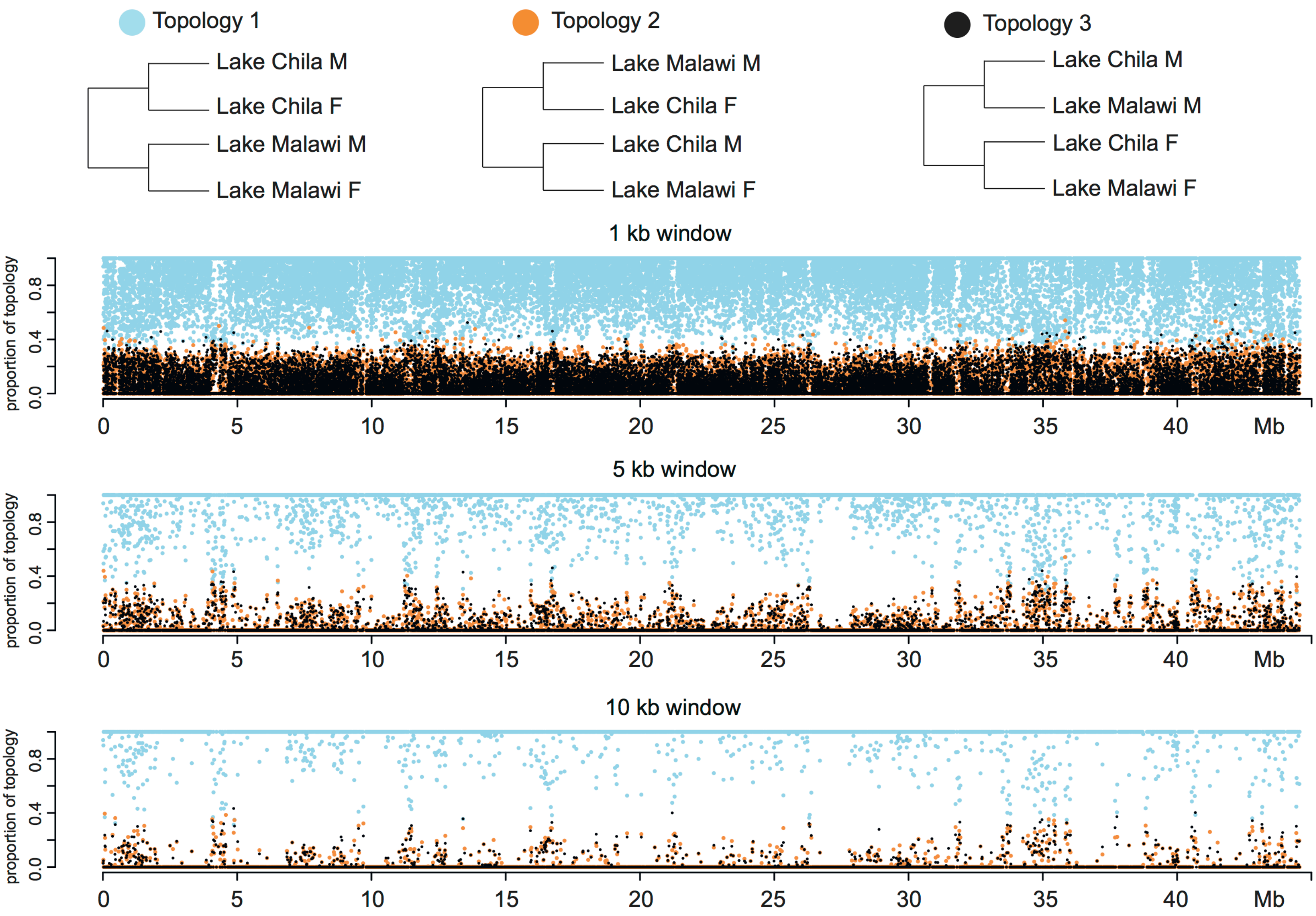

Topology 1
Lake Chila M
Lake Chila F
Lake Malawi M
Lake Malawi F
Topology 2
Lake Malawi M
Lake Chila F
Lake Chila M
Lake Malawi F
Topology 3
Lake Chila M
Lake Malawi M
Lake Chila F
Lake Malawi F
1 kb window
proportion of topology
0.0
0.4
0.8
0
5
10
15
20
25
30
35
40
Mb
5 kb window
10 kb window

Figure S13

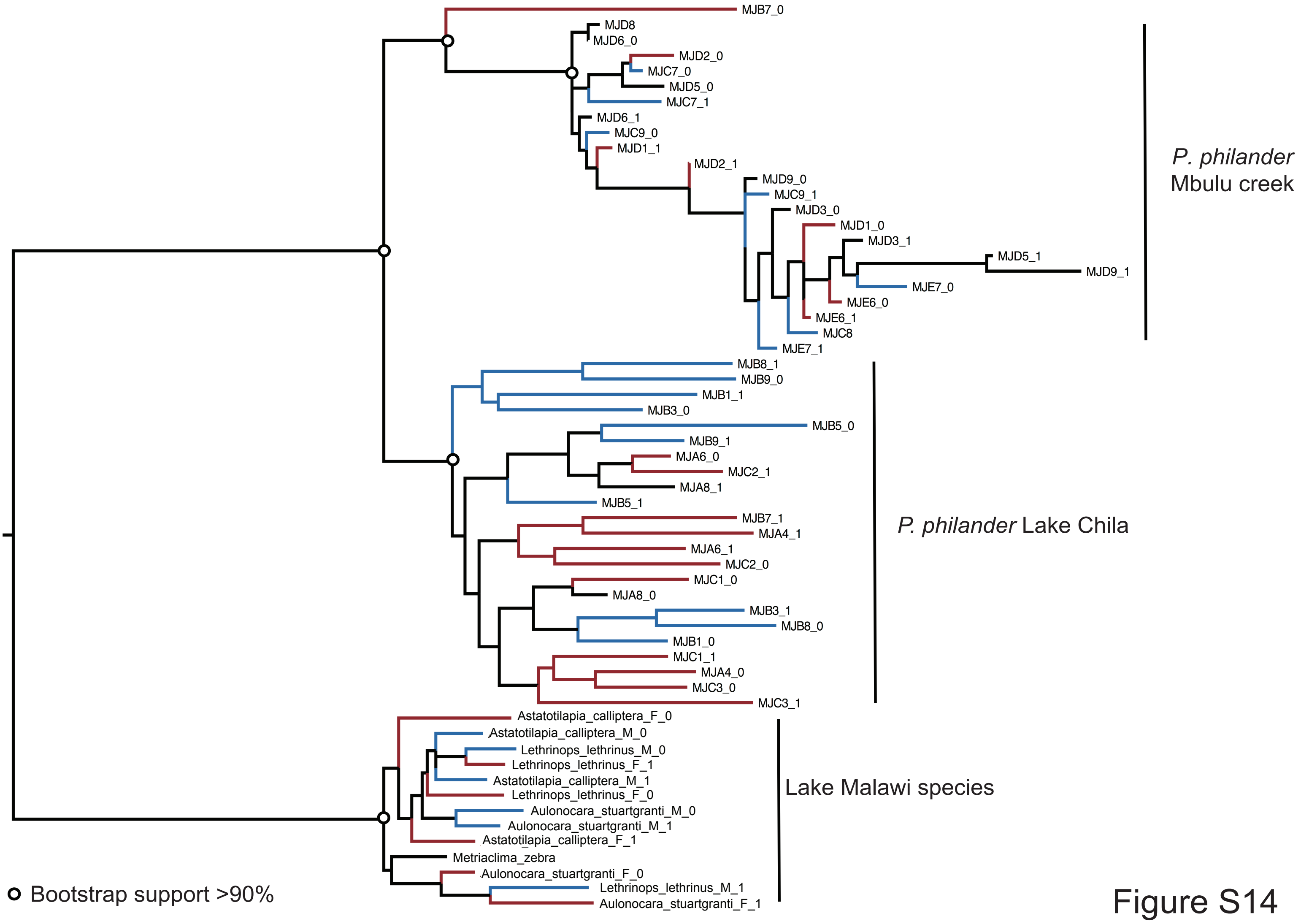

MJB7_0
MJD8
MJD6_0
MJD2_0
MJC7_0
MJD5_0
MJC7_1
MJD6_1
MJC9_0
MJD1_1
MJD2_1
MJD9_0
MJC9_1
MJD3_0
MJD1_0
MJD3_1
MJD5_1
MJD9_1
MJE7_0
MJE6_0
MJE6_1
MJC8
MJE7_1
P. philander
Mbulu creek
MJB8_1
MJB9_0
MJB1_1
MJB3_0
MJB5_0
MJB9_1
MJA6_0
MJC2_1
MJA8_1
MJB5_1
MJB7_1
MJA4_1
MJA6_1
MJC2_0
MJC1_0
MJA8_0
MJB3_1
MJB8_0
MJB1_0
MJC1_1
MJA4_0
MJC3_0
MJC3_1
P. philander Lake Chila
Astatotilapia_calliptera_F_0
Astatotilapia_calliptera_M_0
Lethrinops_lethrinus_M_0
Lethrinops_lethrinus_F_1
Astatotilapia_calliptera_M_1
Lethrinops_lethrinus_F_0
Aulonocara_stuartgranti_M_0
Aulonocara_stuartgranti_M_1
Astatotilapia_calliptera_F_1
Metriaclima_zebra
Aulonocara_stuartgranti_F_0
Lethrinops_lethrinus_M_1
Aulonocara_stuartgranti_F_1
Lake Malawi species
Bootstrap support >90%

Figure S14

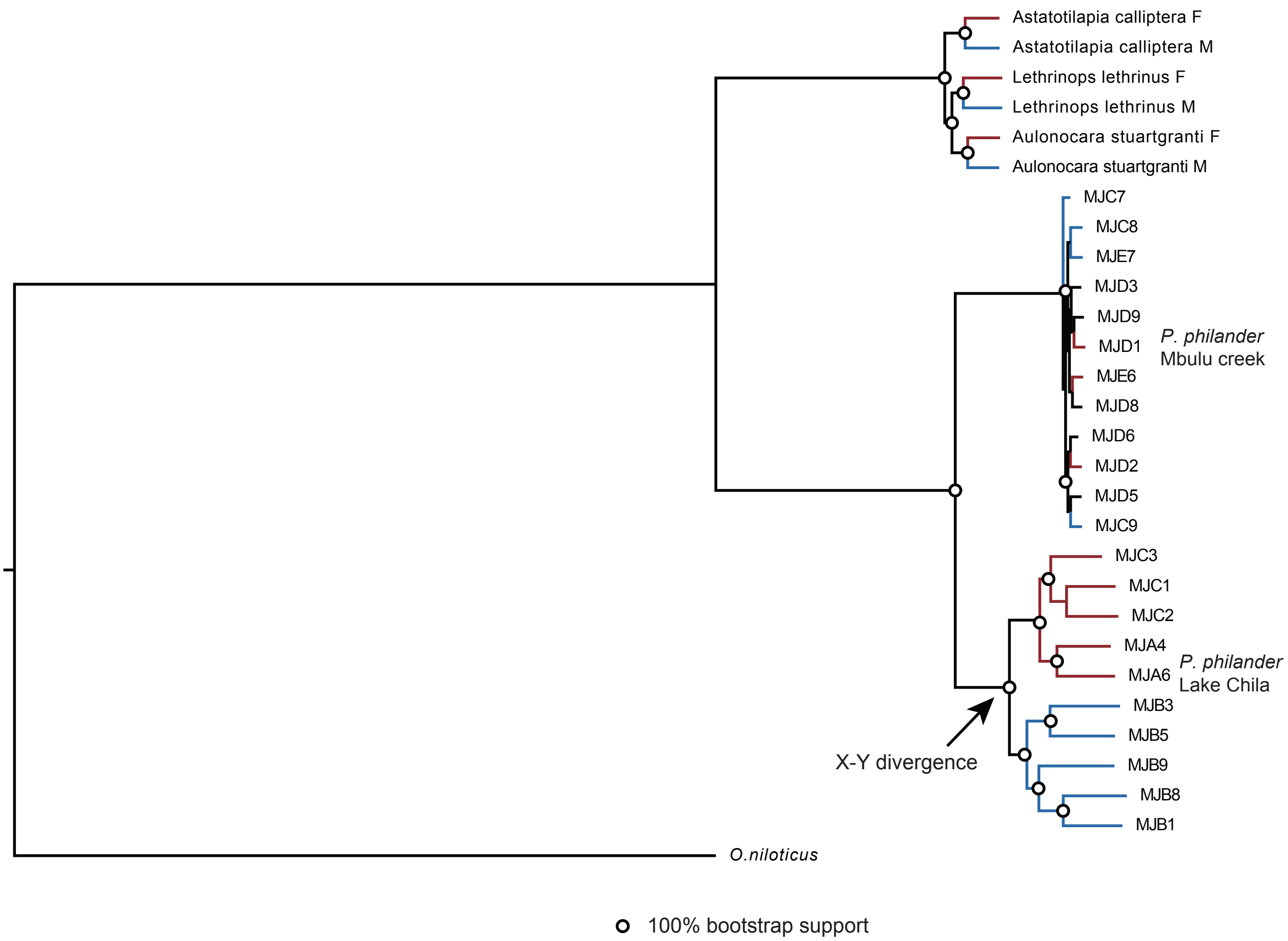
Astatotilapia calliptera F
Astatotilapia calliptera M
Lethrinops lethrinus F
Lethrinops lethrinus M
Aulonocara stuartgranti F
Aulonocara stuartgranti M
MJC7
MJC8
MJE7
MJD3
MJD9
MJD1
MJE6
MJD8
MJD6
MJD2
MJD5
MJC9
P. philander
Mbulu creek
MJC3
MJC1
MJC2
MJA4
MJA6
P. philander
Lake Chila
MJB3
MJB5
MJB9
MJB8
MJB1
X-Y divergence
O.niloticus
100% bootstrap support

Figure S15

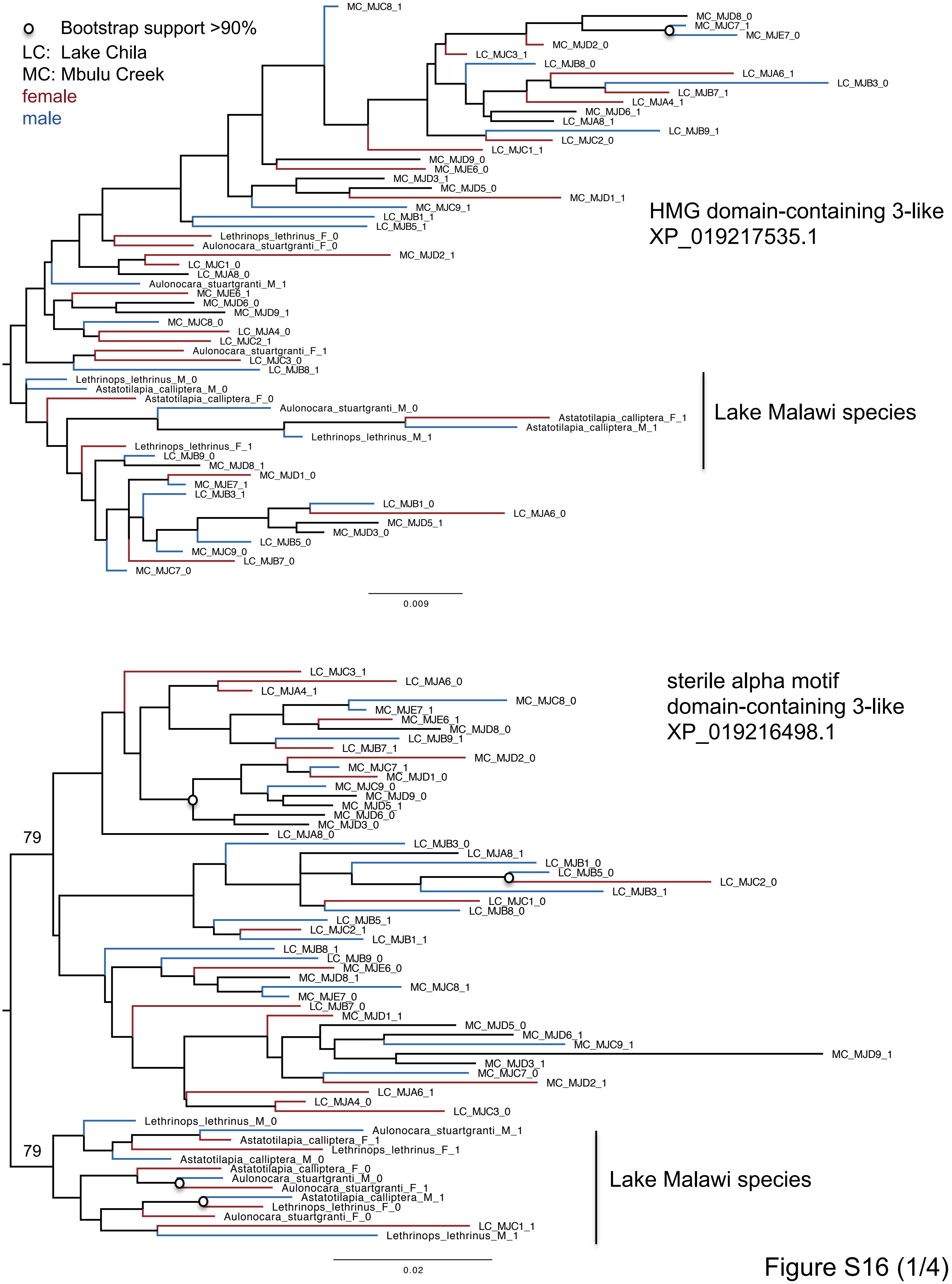

Figure S16 (1/4)

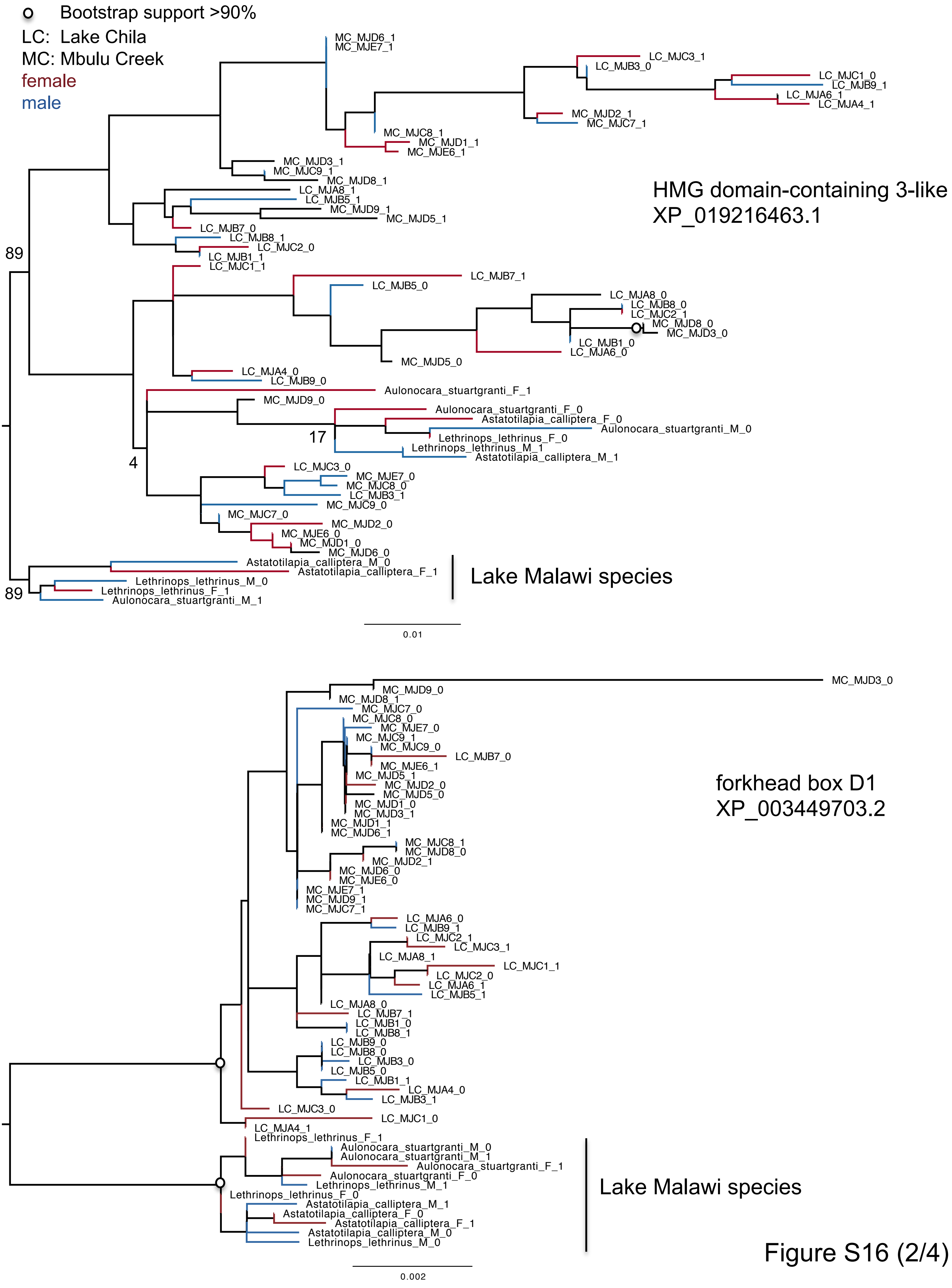

Figure S16 (2/4)

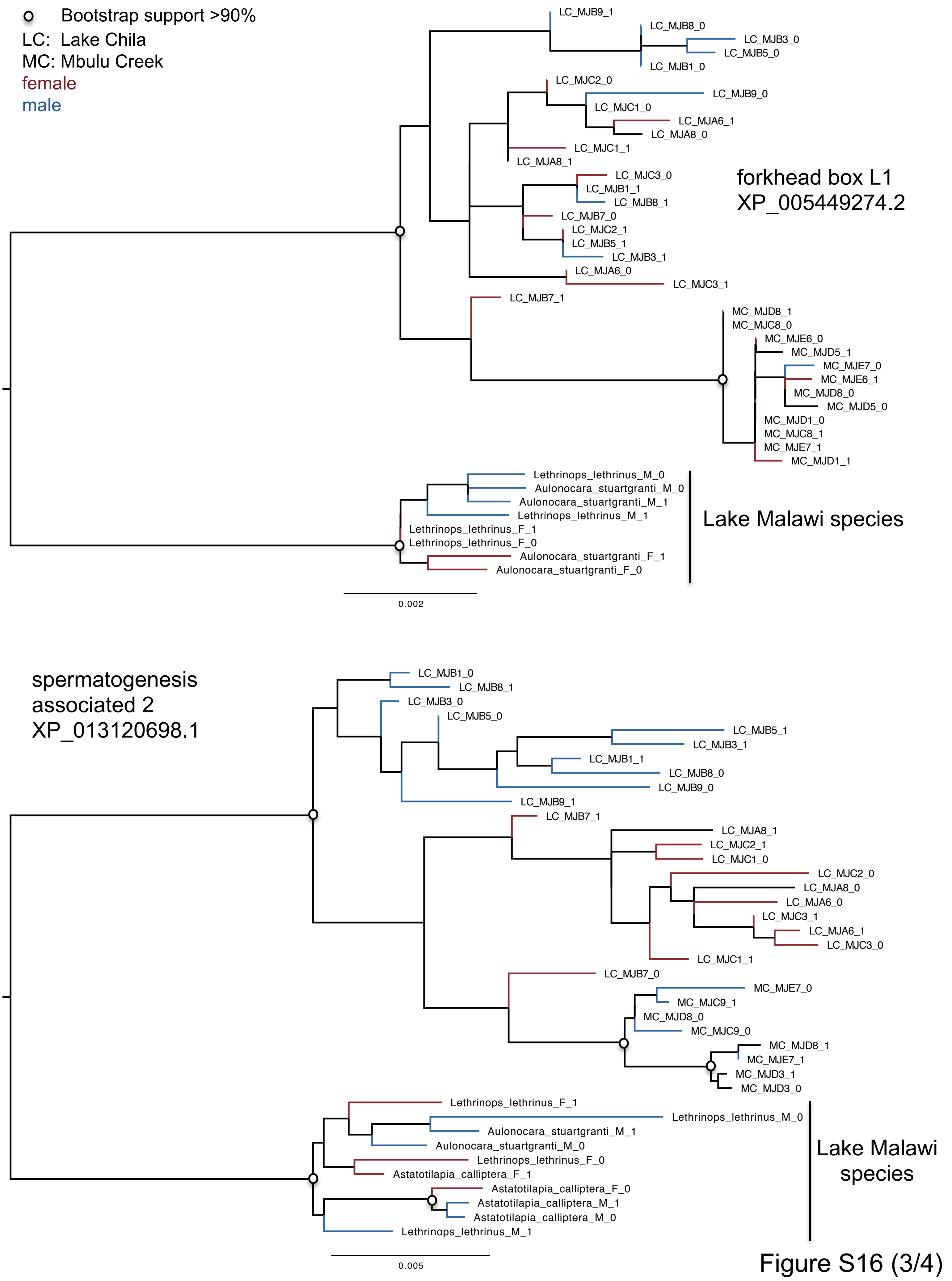

Figure S16 (3/4)

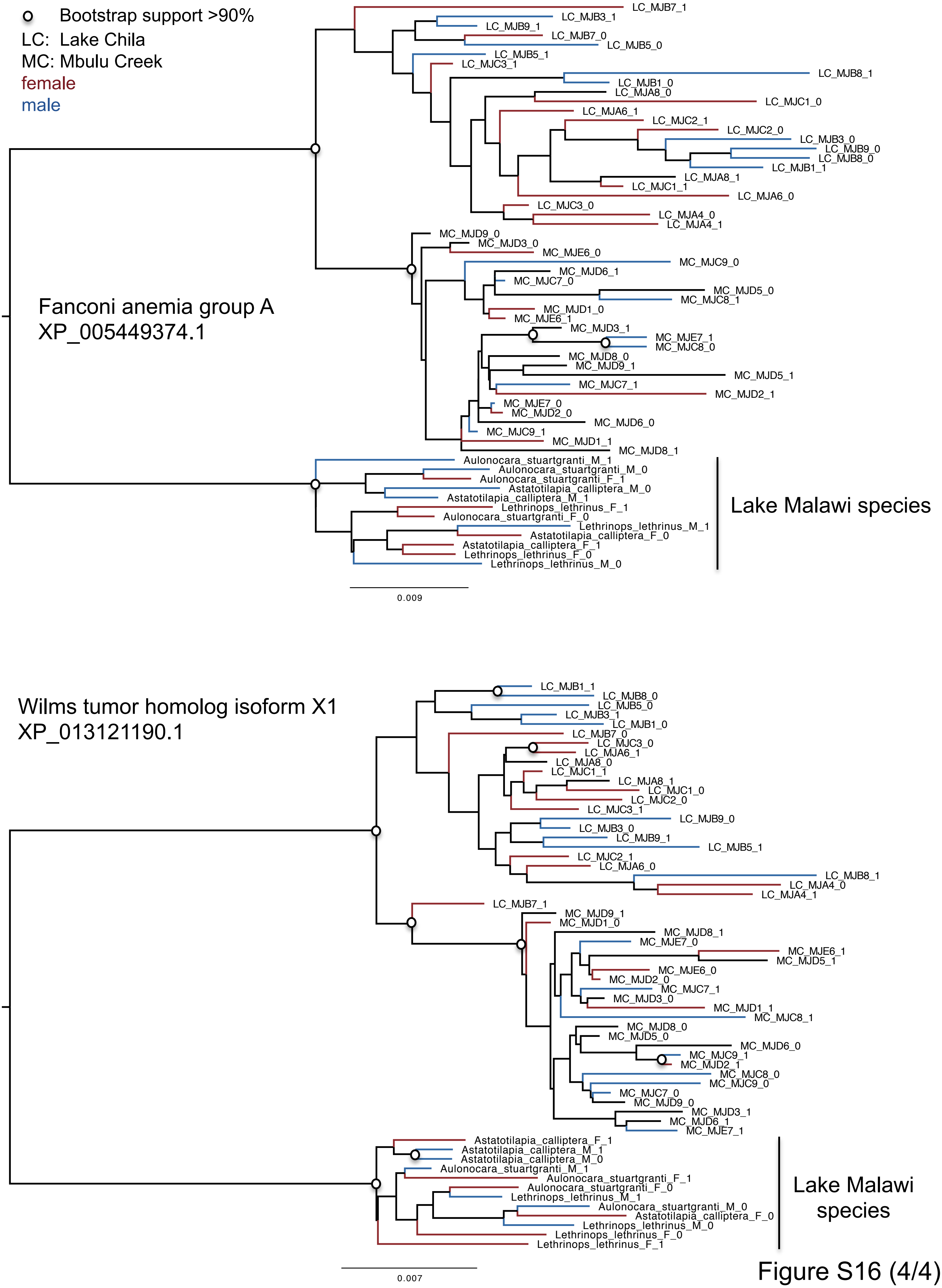

Figure S16 (4/4)

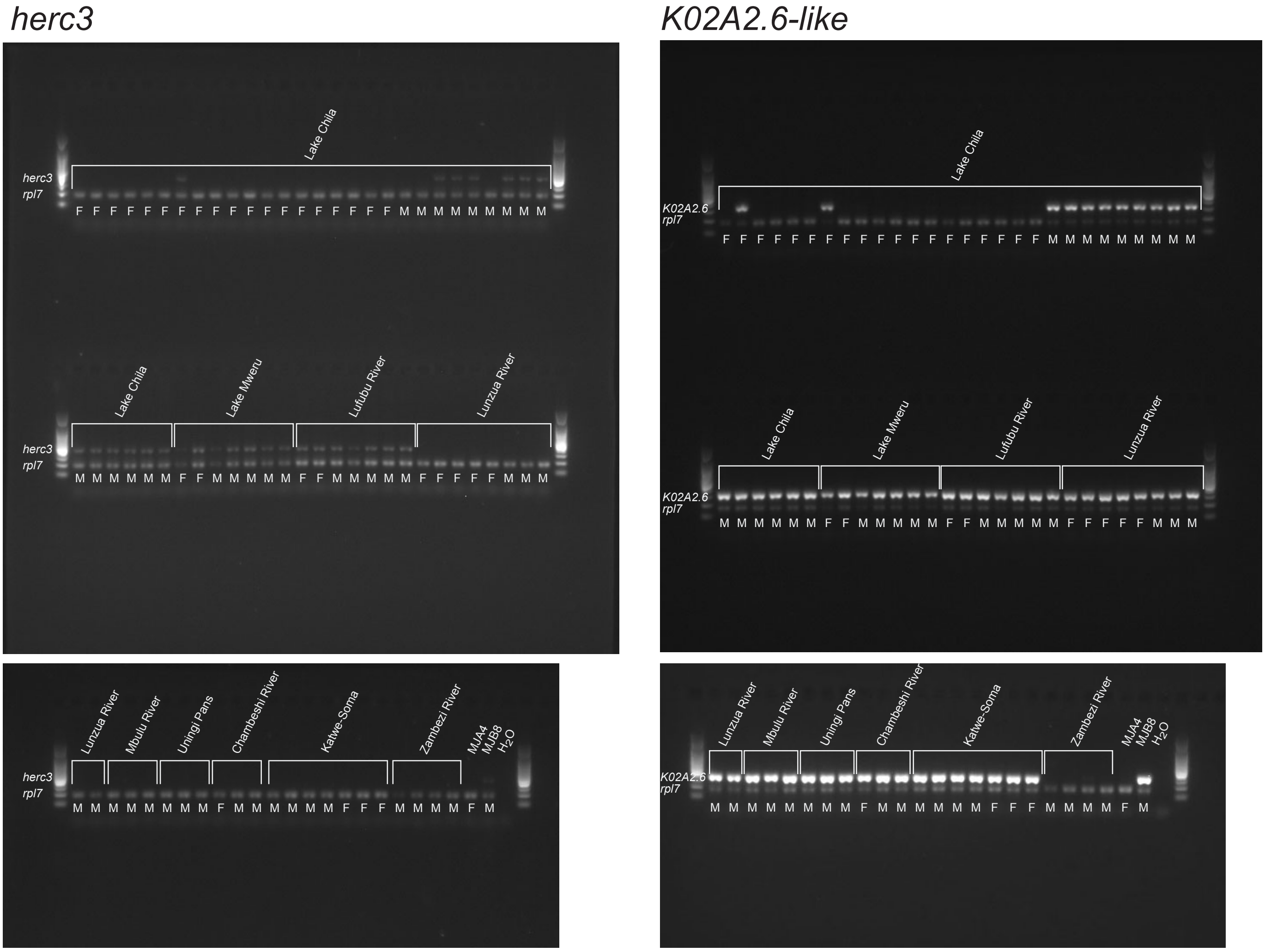
herc3
K02A2.6-like
Lake Chila
herc3
rpl7
K02A2.6
Lake Mweru
Lufubu River
Lunzua River
Mbulu River
Uningi Pans
Chambeshi River
Katwe-Soma
Zambezi River
MJA4
MJB8
H2O

Figure S17
